# dCas12a-mediated CRISPR interference for multiplex gene repression in cyanobacteria for enhanced isobutanol and 3-methyl-1-butanol production

**DOI:** 10.1186/s12934-025-02727-8

**Published:** 2025-05-13

**Authors:** Hao Xie, Barbara Bourgade, Karin Stensjö, Peter Lindblad

**Affiliations:** 1 Microbial Chemistry, Department of Chemistry-Ångström Laboratory, Uppsala University, Uppsala, Sweden; 2 College of Bioengineering, Sichuan University of Science and Engineering, Yibin, Sichuan China; 3 Department of Organismal Biology, Uppsala University, Uppsala, Sweden

**Keywords:** *Synechocystis* sp. PCC 6803, Metabolic engineering, CRISPR interference, Biofuels, Alcohol biosynthesis, Heterologous expression

## Abstract

**Background:**

Cyanobacteria of the genera *Synechocystis* and *Synechococcus* have emerged as promising platforms for metabolic engineering endeavors aimed at converting carbon dioxide into valuable fuels and chemicals, thus addressing the pressing energy demand and mitigating global climate change. Notably, *Synechocystis* sp. strain PCC 6803 (*Synechocystis*) has been engineered to produce isobutanol (IB) and 3-methyl-1-butanol (3M1B) via heterologous expression of α-ketoisovalerate decarboxylase (Kivd). Despite these advances, the achieved IB/3M1B titers remain low. CRISPR interference (CRISPRi), an emerging tool for targeted gene repression, has demonstrated success in various cellular systems to enhance biochemical productivity.

**Results:**

In this study, we developed a dCas12a-mediated CRISPRi system (CRISPRi-dCas12a) that effectively blocked the transcriptional initiation/elongation of essential gene(s), resulting in up to 60% gene repression in *Synechocystis*. Subsequently, the CRISPRi-dCas12a system was successfully integrated into an IB/3M1B producer strain, where it exhibited target gene repression under optimal cultivation conditions. To identify gene targets involved in metabolic pathways potentially limiting IB/3M1B biosynthesis, we initially designed a CRISPR RNA (crRNA) library targeting fifteen individual gene(s), where repression of ten genes significantly increased IB/3M1B production per cell. Moreover, a synergetic effect was observed on IB/3M1B production by designing a single crRNA targeting multiple genes for simultaneous repression. A final strain HX106, featuring dual repression of *ppc* and *gltA*, both involved in the TCA cycle*,* resulted in 2.6-fold and 14.8-fold improvement in IB and 3M1B production per cell, respectively.

**Conclusions:**

Our findings underscore the effectiveness of the CRISPRi-dCas12a system in *Synechocystis* for identifying competing pathways and redirecting carbon flux to enhance IB/3M1B production. Furthermore, this study established a solid groundwork for utilizing an expanded CRISPRi-crRNA library to undertake genome-wide exploration of potential competing pathways not only for IB/3M1B biosynthesis but also for other diverse biofuels and biochemical production processes.

**Supplementary Information:**

The online version contains supplementary material available at 10.1186/s12934-025-02727-8.

## Introduction

A bio-based production of chemicals and fuels from renewable resources is garnering increasing attention, propelled by the growing demand for energy and mounting concerns about global warming stemming from CO_2_ emissions [50]. Cyanobacteria, phototrophic prokaryotes, possess the unique ability to harness sunlight and CO_2_ for photosynthesis. Recent advancements in synthetic biology and metabolic engineering tools have motivated numerous studies exploring the potential of cyanobacteria as green cell factories for producing both commodity and specialized chemicals, such as alcohols [16, 30, 45, 46], terpenes [5], and organic acids [29]. Among these chemicals, isobutanol (IB) emerges as a particularly attractive target chemical with widespread applications, serving as a viable substitute for gasoline due to its versatility, serving as a potential substrate for gasoline, and finding extensive applications in industries ranging from plastics and coating to pharmaceuticals [15].

The initial demonstration of microbial photosynthetic IB production occurred in *Synechococcus elongatus* PCC 7942 (*Synechococcus*), where genes encoding key enzymes including acetolactate synthase (AlsS from *Bacillus subtilis*), acetohydroxy-acid isomeroreductase (IlvC from *Escherichia coli*), dihydroxy-acid dehydratase (IlvD from *E. coli*), α-ketoisovalerate decarboxylase (Kivd from *Lactococcus lactis*), and alcohol dehydrogenases (YqhD from *E. coli*) were heterologously expressed [1]. Subsequently, *Synechocystis* sp. PCC 6803 (*Synechocystis*), another model cyanobacterial strain, was engineered for the production of IB through the sole expression of Kivd [31], resulting in simultaneous production of 3-methyl-1-butanol (3M1B), a preferred gasoline additive and chemical synthesis precursor.

Conventional metabolic engineering strategies have been employed in *Synechocystis* strains for IB and 3M1B production, with enhancements achieved incrementally [33, 45, 47], reaching approximately 150 mg L^−1^ in IB production over 14-day experiments [47]. Leveraging protein engineering as a powerful tool for pathway modification, the key enzyme Kivd underwent targeted modifications. Notably, a single amino acid substitution of Serine286 with Threonine significantly improved Kivd activity, leading to increased IB and 3M1B production [33]. Further investigations involved the expression of multiple copies of *kivd*^*S286T*^ within a single *Synechocystis* cell using two distinct systems: a self-replicating plasmid-based system and a genome integration-based system [45, 47], revealing a positive correlation between IB/3M1B production and *kivd*^*S286T*^ copy numbers. Moreover, elevated IB/3M1B production was achieved by overexpressing enzymes from the 2-keto acid pathway [47] or the central carbon metabolism [45].

Despite recent studies in developing *Synechocystis* as a chassis for IB and 3M1B production by incorporating synthetic pathways or eliminating/downregulating competing pathways, the conventional homologous recombination-based strategy faces challenges, such as time-consuming subsequent cultivation to achieve full segregation, due to the inherent polyploidy of cyanobacteria. The emergence of the clustered regularly interspaced short palindromic repeats-CRISPR-associated protein (CRISPR-Cas) system has revolutionized metabolic engineering in cyanobacteria [6, 38, 41], facilitating targeted and marker-less genome editing with remarkable efficiency [4, 26, 40, 42].

**Fig. 1:**
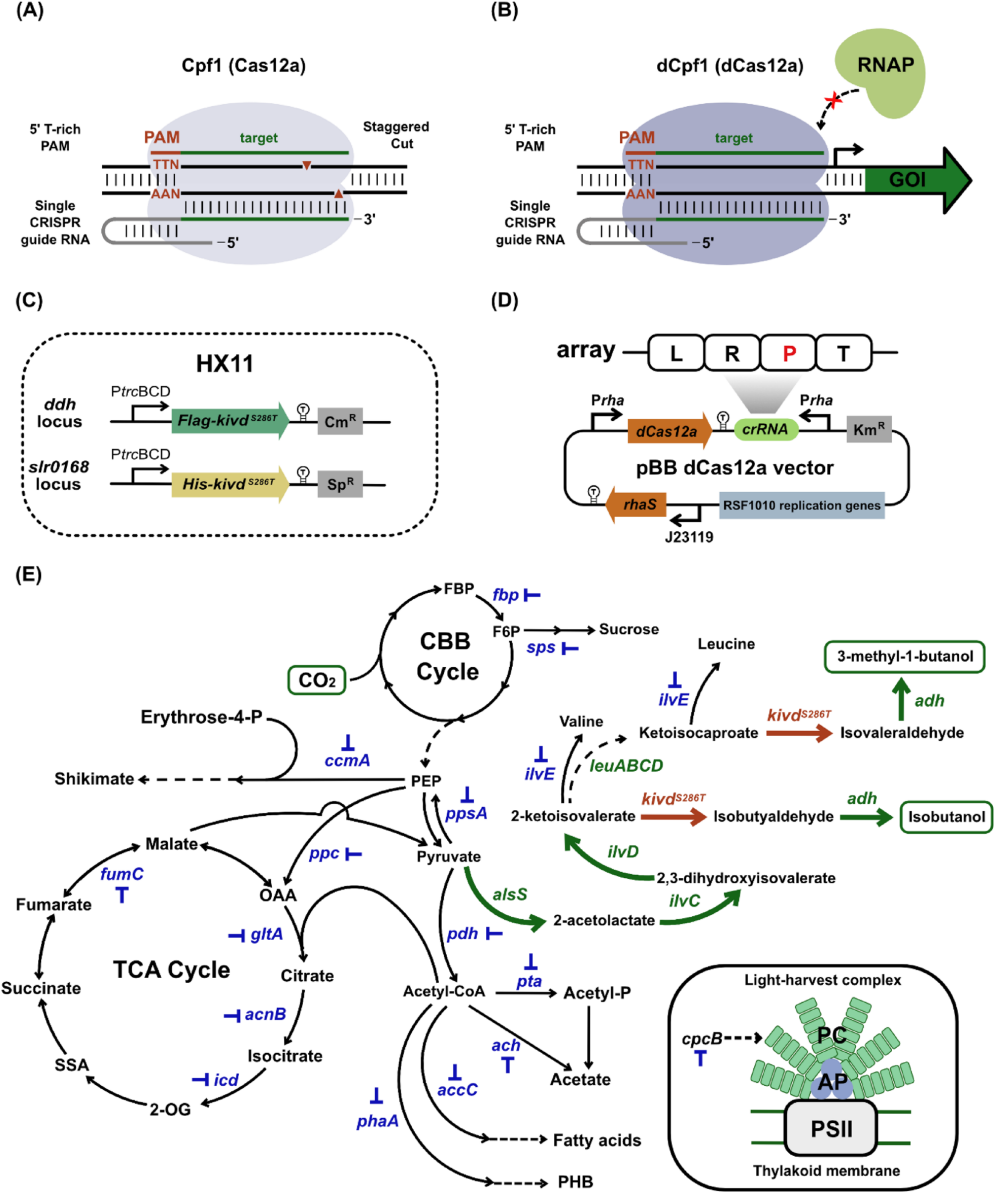
CRISPRi-dCas12a-mediated gene repression in isobutanol (IB)/3-methyl-1-butanol (3M1B) producing *Synechocystis* strain HX11. **A** A scheme of the Cas12a-mediated CRISPR editing system **B** A scheme of the dCas12a-mediated CRISPRi system. **C** Base strain HX11 to test dCas12a-mediated CRISPRi system. Base strain HX11 was constructed by integrating two copies of *kivd*^*S286T*^ into *Synechocystis* genome: Flag-tagged *kivd*^*S286T*^ into the *ddh* locus, and His-tagged *kivd*^*S286T*^ into the *slr0168* locus. **D** pBB_dCas12a vector used for CRISPRi-dCas12a-mediated repression in *Synechocystis*. The vector is developed from a base plasmid pSL3287, harboring a kanamycin resistance cassette and a RSF1010-type replication gene. The expression of dCas12a and CRISPR RNA (crRNA) is driven by P*rha* rhamnose-inducible promoter. The crRNA array consists of four elements: lead sequence, L; direct repeat, R; target specific protospacer region, P; terminator, T. **E** Schematics of the metabolic pathways for photosynthetic IB/3M1B production in *Synechocystis* and selected genes for repression using dCas12a-mediated CRISPRi. Gene names in blue represent the repression targets; gene names in green represent the endogenous enzymes of 2-keto acid pathway for IB/3M1B production; gene name in red represents the heterologous enzyme of 2-keto acid pathway for IB/3M1B production. *accC* acetyl-CoA carboxylase, *ach* acetyl-CoA hydrolase, *acnB* aconitase, *adh* alcohol dehydrogenase, *alsS* acetolactate synthase, *ccmA* 3-Deoxy-D-arabinoheptulosonate 7-phosphate (DAHP) synthase, *cpcB* phycocyanin β-subunit, *fbp* fructose 1,6-bisphosphatase, *fumC* fumarase, *gltA* citrate synthase, *icd* isocitrate dehydrogenase, *ilvC* acetohydroxy-acid isomeroreductase, *ilvD* dihydroxy-acid dehydratase, *ilvE* branched-chain amino acid aminotransferase, *kivd*^*S286T*^ α-ketoisovalerate decarboxylase, *leuA* 2-isopropylmalate synthase, *leuB* 3-isopropylmalate dehydrogenase, *leuCD* 3-isopropylmalate dehydratase, *ppc* phosphoenolpyruvate (PEP) carboxylase, *pdh* pyruvate dehydrogenase, *phaA* acetyl-CoA acetyltransferase, *ppsA* phosphoenolpyruvate synthase, *pta* phosphotransacetylase, *sps* sucrose phosphate synthase, *FBP* fructose-1,6-bisphosphate, *F6P* fructose-6-phosphate, *OAA* oxaloacetate, *PEP* phosphoenolpyruvate, *PHB* poly-3-hydroxybutyrate, *SSA* succinate semialdehyde, *2-OG* 2-oxoglutarate, *AP* allophycocyanin, *PC* phycocyanin, *PSII* photosystem II

Derived from the CRISPR-Cas system, the CRISPR interference-deactivated Cas (CRISPRi-dCas) system, lacking cleavage activity while retaining the ability to bind to target DNA using a single guide RNA, stands out as a preferred tool for fine-tuning gene expression [36]. Operating through a deactivated form of the Cas9 nuclease (dCas9), the CRISPRi-dCas9 system achieves controllable gene expression by sterically repressing transcription, either through blocking transcriptional initiation or elongation. This positions it as a valuable tool for manipulating metabolic pathways to optimize bioproduction. Experimental demonstrations have showcased its effectiveness in increasing production of various compounds in cyanobacteria, such as 2,3-butanediol [25], succinate [19], octadecanol [20], and extracellular polymeric substances (EPS) [37]. Addressing toxicity concerns associated with the Cas9 protein in cyanobacteria [42], the Cas protein Cpf1 (renamed as Cas12a), a type V-A nuclease from the class II family of CRISPR systems originating from *Francisella novicida* [48], has been identified as non-toxic in cyanobacteria. Unlike Cas9, Cas12a is a dual-nuclease responsible for both CRISPR RNA (crRNA) maturation and target DNA interference [13], making it suitable for multiplex genome editing. Utilizing a T-rich protospacer-adjacent motif (PAM), the mature crRNA guides Cas12a to recognize and cut the target DNA, generating a double-stranded break with a 5’ overhang [13] (Fig. 1A). Consequently, Cas12a can be converted to a deactivated form, dCas12a, via a single D917A mutation [48] (Fig. 1B), which retains the ability to bind to target DNA sequence strongly, sufficient to repress gene transcription. The established CRISPRi-dCas12a system has been effective in tunable repression of key photosynthetic processes in *Synechocystis* [28] and *Synechococcus elongatus* UTEX 2973 [24]. It was further applied in *Synechococcus* to enhance squalene production by repressing *cpcB* and *acnB* genes [9]. However, this system has yet to be exploited in *Synechocystis* for bioproduction enhancement.

In this study, we developed a dCas12a-mediated CRISPR interference (CRISPRi-dCas12a) system that effectively blocked the transcriptional initiation/elongation of essential gene(s). We subsequently applied this tool to enhance IB and 3M1B production by systematically evaluating potential competing pathways in a *Synechocystis* strain engineered for IB and 3M1B production. We targeted genes involved in photosynthesis efficiency, carbon storage compound synthesis, and those relevant to consumption of IB/3M1B precursors, such as pyruvate or 2-ketoisovalerate. Before systematically characterizing the effects of repressing the proposed fifteen gene targets, we optimized cultivation condition to ensure suitability for testing the CRISPRi-dCas12a system in the IB/3M1B-producing *Synechocystis* strain. Repression of ten target genes—*ccmA*, *ppsA*, *ppc*, *gltA*, *acnB*, *accC*, *pdh*, *cpcB*, *ilvE*, and *sps*—resulted in significantly improved IB and/or 3M1B production per cell. Subsequently, combined inhibition of these effective targets was tested, and a synergetic effect was observed. Our findings present a novel application of CRISPRi-dCas12a system in *Synechocystis* for systematically and rapidly mapping potential competing pathways for IB/3M1B biosynthesis. The identified targets offer valuable insights for enhancing pyruvate-derived chemical bioproduction in various cyanobacterial species.

## Material and methods

### Strains and growth conditions

*Escherichia coli* (*E. coli*) strain DH5α-Z1 (Invitrogen) was employed for cloning purpose, while *E. coli* strain HB101 containing plasmid pRL443-AmpR was utilized for conjugation. Cultures were maintained at 37 °C in lysogeny broth (LB) medium (Sigma-Aldrich), supplemented with appropriate antibiotics (50 μg mL^−1^ kanamycin, 100 μg mL^−1^ ampicillin). Glucose-tolerant *Synechocystis* sp. PCC 6803 strain was employed throughout the study. *Synechocystis* seed cultures were cultivated and propagated under conditions of 30 μmol photons m^−2^ s^−1^ at 30 °C in BG11 medium. All *Synechocystis* strains used in this study are listed in Table 1.

**Table 1:** *Synechocystis* strains used in this study

Strain	Relevant genotypes^a^	References
WT	Wild-type *Synechocystis* sp. PCC 6803	[43]
BB1	pBB_P*rha*_***dCas12a***_T_P*rha*_***crRNA(pdhB)***_Km^R^	This study
BB2	pBB_P*rha*_***dCas12a***_T_P*rha*_***crRNA(dxs)***_Km^R^	This study
BB3	pBB_P*rha*_***dCas12a***_T_P*rha*_***crRNA(no target)***_Km^R^	This study
HX11	*Δddh*::(P*trc*BCD-***kivd***^***S286T***^-T)-Cm^R^, *Δslr0168*::(P*trc*BCD-***kivd***^***S286T***^-T)-Sp^R^	This study
HX11-pEEK*	*Δddh*::(P*trc*BCD-***kivd***^***S286T***^-T)-Cm^R^, *Δslr0168*::(P*trc*BCD-***kivd***^***S286T***^-T)-Sp^R^, pEEK*	This study
HX11-EVC	*Δddh*::(P*trc*BCD-***kivd***^***S286T***^-T)-Cm^R^, *Δslr0168*::(P*trc*BCD-***kivd***^***S286T***^-T)-Sp^R^, pBB_P*rha*_***dCas12a***_T_P*rha*_***crRNA(no target)***_Km^R^	This study
HX11-accC	*Δddh*::(P*trc*BCD-***kivd***^***S286T***^-T)-Cm^R^, *Δslr0168*::(P*trc*BCD-***kivd***^***S286T***^-T)-Sp^R^, pBB_P*rha*_***dCas12a***_T_P*rha*_***crRNA(accC)***_Km^R^	This study
HX11-acnB	*Δddh*::(P*trc*BCD-***kivd***^***S286T***^-T)-Cm^R^, *Δslr0168*::(P*trc*BCD-***kivd***^***S286T***^-T)-Sp^R^, pBB_P*rha*_***dCas12a***_T_P*rha*_***crRNA(acnB)***_Km^R^	This study
HX11-ccmA	*Δddh*::(P*trc*BCD-***kivd***^***S286T***^-T)-Cm^R^, *Δslr0168*::(P*trc*BCD-***kivd***^***S286T***^-T)-Sp^R^, pBB_P*rha*_***dCas12a***_T_P*rha*_***crRNA(ccmA)***_Km^R^	This study
HX11-cpcB	*Δddh*::(P*trc*BCD-***kivd***^***S286T***^-T)-Cm^R^, *Δslr0168*::(P*trc*BCD-***kivd***^***S286T***^-T)-Sp^R^, pBB_P*rha*_***dCas12a***_T_P*rha*_***crRNA(cpcB)***_Km^R^	This study
HX11-fbp_glpX	*Δddh*::(P*trc*BCD-***kivd***^***S286T***^-T)-Cm^R^, *Δslr0168*::(P*trc*BCD-***kivd***^***S286T***^-T)-Sp^R^, pBB_P*rha*_***dCas12a***_T_P*rha*_***crRNA(fbp, glpX)***_Km^R^	This study
HX11-fumC	*Δddh*::(P*trc*BCD-***kivd***^***S286T***^-T)-Cm^R^, *Δslr0168*::(P*trc*BCD-***kivd***^***S286T***^-T)-Sp^R^, pBB_P*rha*_***dCas12a***_T_P*rha*_***crRNA(fumC)***_Km^R^	This study
HX11-gltA	*Δddh*::(P*trc*BCD-***kivd***^***S286T***^-T)-Cm^R^, *Δslr0168*::(P*trc*BCD-***kivd***^***S286T***^-T)-Sp^R^, pBB_P*rha*_***dCas12a***_T_P*rha*_***crRNA(gltA)***_Km^R^	This study
HX11-icd	*Δddh*::(P*trc*BCD-***kivd***^***S286T***^-T)-Cm^R^, *Δslr0168*::(P*trc*BCD-***kivd***^***S286 T***^-T)-Sp^R^, pBB_P*rha*_***dCas12a***_T_P*rha*_***crRNA(icd)***_Km^R^	This study
HX11-ilvE	*Δddh*::(P*trc*BCD-***kivd***^***S286T***^-T)-Cm^R^, *Δslr0168*::(P*trc*BCD-***kivd***^***S286T***^-T)-Sp^R^, pBB_P*rha*_***dCas12a***_T_P*rha*_***crRNA(ilvE)***_Km^R^	This study
HX11-pdh	*Δddh*::(P*trc*BCD-***kivd***^***S286T***^-T)-Cm^R^, *Δslr0168*::(P*trc*BCD-***kivd***^***S286T***^-T)-Sp^R^, pBB_P*rha*_***dCas12a***_T_P*rha*_***crRNA(sll1721, slr1934)***_Km^R^	This study
HX11-phaA	*Δddh*::(P*trc*BCD-***kivd***^***S286T***^-T)-Cm^R^, *Δslr0168*::(P*trc*BCD-***kivd***^***S286T***^-T)-Sp^R^, pBB_P*rha*_***dCas12a***_T_P*rha*_***crRNA(phaA)***_Km^R^	This study
HX11-ppc	*Δddh*::(P*trc*BCD-***kivd***^***S286T***^-T)-Cm^R^, *Δslr0168*::(P*trc*BCD-***kivd***^***S286T***^-T)-Sp^R^, pBB_P*rha*_***dCas12a***_T_P*rha*_***crRNA(ppc)***_Km^R^	This study
HX11-ppsA	*Δddh*::(P*trc*BCD-***kivd***^***S286T***^-T)-Cm^R^, *Δslr0168*::(P*trc*BCD-***kivd***^***S286T***^-T)-Sp^R^, pBB_P*rha*_***dCas12a***_T_P*rha*_***crRNA(ppsA)***_Km^R^	This study
HX11-pta_ach	*Δddh*::(P*trc*BCD-***kivd***^***S286T***^-T)-Cm^R^, *Δslr0168*::(P*trc*BCD-***kivd***^***S286T***^-T)-Sp^R^, pBB_P*rha*_***dCas12a***_T_P*rha*_***crRNA(pta, ach)***_Km^R^	This study
HX11-sps	*Δddh*::(P*trc*BCD-***kivd***^***S286T***^-T)-Cm^R^, *Δslr0168*::(P*trc*BCD-***kivd***^***S286T***^-T)-Sp^R^, pBB_P*rha*_***dCas12a***_T_P*rha*_***crRNA(sps)***_Km^R^	This study
HX106	*Δddh*::(P*trc*BCD-***kivd***^***S286T***^-T)-Cm^R^, *Δslr0168*::(P*trc*BCD-***kivd***^***S286T***^-T)-Sp^R^, pBB_P*rha*_***dCas12a***_T_P*rha*_***crRNA(ppc, gltA)***_Km^R^	This study
HX107	*Δddh*::(P*trc*BCD-***kivd***^***S286T***^-T)-Cm^R^, *Δslr0168*::(P*trc*BCD-***kivd***^***S286T***^-T)-Sp^R^, pBB_P*rha*_***dCas12a***_T_P*rha*_***crRNA(ppc, gltA, acnB)***_Km^R^	This study
HX108	*Δddh*::(P*trc*BCD-***kivd***^***S286T***^-T)-Cm^R^, *Δslr0168*::(P*trc*BCD-***kivd***^***S286T***^-T)-Sp^R^, pBB_P*rha*_***dCas12a***_T_P*rha*_***crRNA(ppc, gltA, ppsA)***_Km^R^	This study
HX109	*Δddh*::(P*trc*BCD-***kivd***^***S286T***^-T)-Cm^R^, *Δslr0168*::(P*trc*BCD-***kivd***^***S286T***^-T)-Sp^R^, pBB_P*rha*_***dCas12a***_T_P*rha*_***crRNA(ppc, gltA, cpcB)***_Km^R^	This study
HX110	*Δddh*::(P*trc*BCD-***kivd***^***S286T***^-T)-Cm^R^, *Δslr0168*::(P*trc*BCD-***kivd***^***S286T***^-T)-Sp^R^, pBB_P*rha*_***dCas12a***_T_P*rha*_***crRNA(ppc, gltA, accC)***_Km^R^	This study
HX111	*Δddh*::(P*trc*BCD-***kivd***^***S286T***^-T)-Cm^R^, *Δslr0168*::(P*trc*BCD-***kivd***^***S286T***^-T)-Sp^R^, pBB_P*rha*_***dCas12a***_T_P*rha*_***crRNA(ppc, ppsA, ccmA)***_Km^R^	This study
HX112	*Δddh*::(P*trc*BCD-***kivd***^***S286T***^-T)-Cm^R^, *Δslr0168*::(P*trc*BCD-***kivd***^***S286T***^-T)-Sp^R^, pBB_P*rha*_***dCas12a***_T_P*rha*_***crRNA(ppsA, accC, sll1721, slr1934)***_Km^R^	This study

Expressed *genes* in bold

^a^Km^R^ Kanamycin resistance cassette, Sp^R^ spectinomycin resistance cassette, Cm^R^ chloramphenicol resistance cassette, T Terminator BBa_B0015

### Plasmid construction

The plasmids employed and constructed in this study are detailed in Table S1. Plasmids pHX8 (pDdh_(P*trc*BCD_***kivd***^***S286T***^_T)_Cm^R^) and pHX15 (pSlr0168_(P*trc*BCD_***kivd***^***S286T***^_T)_Sp^R^) were previously constructed in a separate study (Fig. 1C) [47]. Derived from the pSL3287 plasmid (Addgene; #139750) [24], a foundational CRISPRi plasmid, pBB_dCas12a, was developed. This plasmid expressed dCas12a and CRISPR RNA (crRNA) under a rhamnose-inducible P*rha* promoter (Fig. 1D) [21]. RhaS and P*rha* were amplified from plasmid pSHDY-Prha-mVenus-rhaS [3] (a kind gift from Professor I. Axmann’s group (Heinrich-Heine-Universität Düsseldorf, Germany)) using primer pairs RhaS_F/R, Prha_F/R, and Prha_gRNA_F/R (Table S2). Both fragments were cloned into pSL3287. Target(s)-specific CRISPRi plasmids were generated by cloning annealed oligonucleotides (Table S2) into the AarI sites of pBB_dCas12a plasmid, as previously described [40]. All pBB_dCas12a and pBB_dCas12a-derived CRISPRi plasmids used in this study feature an RSF1010-type replicon and mobilization region [39].

### Cyanobacteria strain construction

The foundational strain for isobutanol (IB) and 3-methyl-1-butanol (3M1B) production, HX11, was established through double homologous recombination via natural transformation, as previously outlined (Fig. 1C, and Table 1) [47]. Target-specific CRISPRi plasmids were introduced into the wild-type strain or HX11 strain individually through triparental mating [18], employing *E. coli* strain HB101 carrying the conjugative plasmid pRL443-AmpR. A detailed conjugation protocol is available in [47]. Single colonies were subsequently transferred to BG11 agar plates supplemented with 50 µg mL^−1^ kanamycin (for wild-type background) or 10 µg mL^−1^ chloramphenicol, 25 µg mL^−1^ spectinomycin, and 25 µg mL^−1^ kanamycin (for HX11 background). Positive colonies were verified through colony PCR using target-specific primers (Table S2) and DreamTaq DNA polymerase (Thermo Scientific). For each conjugation event, three confirmed positive colonies were retained for further analysis. A comprehensive list of all strains employed and constructed in this study is provided in Table 1.

### Cultivation condition for CRISPRi-dCas12a characterization

*Synechocystis* seed cultures were incubated at 30 °C in 6-well plates (SARSTEDT) under continuous white light (30 μmol photons m^−2^ s^−1^) in BG11 medium supplemented with 50 µg mL^−1^ kanamycin. From these seed cultures, 25 mL of BG11 medium supplemented with 50 µg mL^−1^ kanamycin and 3 mM of L-rhamnose (hereafter rhamnose), unless stated otherwise, were inoculated in 100 mL Erlenmeyer flasks (VWR) as experimental cultures at an initial optical density (OD_750_) of 0.1, unless stated otherwise. Experimental cultures were incubated at 30 °C under continuous white light (50 μmol photons m^−2^ s^−1^) and shaken at 120 rpm. For transcript quantification, cells were collected by centrifugation at 4,500 xg for 5 min and washed once with sterile H_2_O. Cell pellets were stored at −80 °C until RNA extraction. All cultures were performed in three biological replicates. CRISPRi-targeted strains were compared to strain BB3 (Table 1) carrying pBB_dCas12a (Table S1).

### Cultivation condition for IB and 3M1B production

*Synechocystis* seed cultures were incubated at 30 °C in 100 mL Erlenmeyer flasks (VWR) under continuous white light (30 μmol photons m^−2^ s^−1^) in BG11 medium supplemented with 10 µg mL^−1^ chloramphenicol, 25 µg mL^−1^ spectinomycin, and 25 µg mL^−1^ kanamycin. These seed cultures were used to inoculate 25 ml experimental cultures in BioLite 25 cm^2^ plug-sealed tissue culture flasks (Thermo Scientific) with an initial OD_750_ of 0.1. The medium for experimental cultures was BG11 containing 50 mM NaHCO_3_ (Sigma-Aldrich), 10 µg mL^−1^ chloramphenicol, 25 µg mL^−1^ spectinomycin, and 25 µg mL^−1^ kanamycin. The experimental cultures were horizontally shaken at 120 rpm under continuous white light (50 μmol photons m^−2^ s^−1^) at 30 °C.

#### Initial validation of CRISPRi-dCas12a in strain HX11

All CRISPRi-targeted strains were compared to the control strain HX11-EVC (Table 1) during IB/3M1B experiments. All experimental cultures were conducted in technical duplicates. On day 2, 3 mM rhamnose were added to the culture medium for induction. Every second day, 2 mL of culture was replaced with 2 mL of fresh BG11 medium, supplemented with 500 mM NaHCO_3_, 3 mM rhamnose, and appropriate antibiotics. OD_750_ was measured daily, while IB and 3M1B production were measured on days 3, 4, and 5. The cultivation period extended for 5 days. For transcript quantification, cells were collected on day 4 by centrifugation at 4,500 xg for 5 min and washed once with sterile H_2_O. Cell pellets were stored at −80 °C until RNA extraction.

#### Examination the effectiveness of three conditions on CRISPRi-dCas12a system in strain HX11

All experimental cultures were conducted in biological triplicates and inoculated at an initial OD_750_ of 0.1. To examine different rhamnose induction timepoints, 3 mM rhamnose were added at various stages: in the seed culture before inoculation, on days 0, 1, 2, or 3. For different rhamnose concentrations, cultures were induced on day 1 with concentrations of 1 mM, 3 mM, 5 mM, or 10 mM. For HCl-titrated cultures, 37% HCl was added daily to maintain a pH between 7 and 8. Culture pH was monitored using MColorpHast^™^ pH-indicator strips (pH 6.5–10) (Merck). Every second day, 2 mL of culture was replaced with 2 mL of fresh BG11 medium supplemented with 500 mM NaHCO_3_ and appropriate antibiotics. OD_750_ was measured daily, while IB and 3M1B production were measured on days 2, 3, 4, and 5. The cultivation period lasted for 5 days.

#### Characterizing engineered strains at optimized cultivation condition

All experimental cultures were conducted in biological triplicates. On day 0, 3 mM rhamnose were added for induction, followed by replacement of 2 mL of culture with 2 mL of fresh BG11 medium containing 500 mM NaHCO_3_ and appropriate antibiotics every second day. Daily measurements of OD_750_ were taken, and IB and 3M1B production were measured on days 2, 3, and 4. The cultivation duration was 4 days.

### Optical density measurement

Cell growth was assessed by measuring the optical density at a wavelength of 750 nm (OD_750_), following previously described protocol [47].

### Products analysis

IB and 3M1B were extracted from the culture supernatant using dichloromethane as the extraction solvent, following the procedures outlined in previous work [47]. Quantification of the extracted IB and 3M1B was conducted using gas chromatography (GC), as detailed previously [47]. In summary, a PerkinElmer GC 580 system equipped with a flame ionization detector and an Elite-WAX Polyethylene Glycol Series Capillary column (30 m × 0.25 mm × 0.25 μm, PerkinElmer) was employed for quantification. Nitrogen served as the carrier gas at a rate of 10 mL min^−1^. The injector and detector temperatures were set at 220 °C and 240 °C, respectively. The GC results obtained were analyzed using TotalChrom Navigator version 6.3.2.

### Real-time quantitative PCR (RT-qPCR)

The frozen cell pellets were resuspended in 500 µl TRI Reagent^®^ (Sigma-Aldrich). Following the addition of 0.2 g acid-washed glass beads (425–600 μm diameter, Sigma Aldrich), cell disruption was performed using the Precellys-24 Beadbeater (Bertin Instruments) with the following program: 5,000 rpm program 4 × 30 s, with a 2-min incubation on ice between runs. The extraction mixture was centrifuged at 18,213 xg for 10 min at 4 °C to separate the supernatant, which was then transferred into a new Eppendorf tube. Subsequently, spin-column total RNA purification was carried out using the Direct-zol™ RNA Miniprep kit (Zymo Research), following the manufacturer's recommendations, excluding the DNase I treatment step. The purified total RNA underwent two rounds of DNase I treatment (Thermo Fisher Scientific) to eliminate potential genomic DNA, followed by another spin-column total RNA purification using the Direct-zol^™^ RNA Miniprep kit (Zymo Research). The concentration of the purified total RNA was measured using a Nanodrop^™^ 2000 spectrophotometer (Thermo Scientific). For cDNA synthesis, 500 ng of total RNA was utilized with the iScript cDNA synthesis kit (BIO-RAD). RT-qPCR was conducted using the iTaq Universal SYBR Green Supermix (BIO-RAD) on a CFX Connect Real-Time PCR system (BIO-RAD), following the manufacturer’s recommendations. Previously tested primers [35] for the *rnpB* gene, encoding the RNA subunit of ribonuclease P, were used for the internal control. The primers employed for RT-qPCR are detailed in Table S2.

### Crude protein extraction and SDS-PAGE/Western-immunoblot

Crude protein extraction and SDS-PAGE/Western-immunoblot was performed as previously detailed [47]. Twenty micrograms (His-tagged and Flag-tagged proteins) of soluble crude proteins were loaded for protein expression analysis.

## Results and discussion

### Initial design and characterization of CRISPRi-dCas12a system in *Synechocystis*

Cas12a, previously known as Cpf1, sourced from *Francisella novicida*, has emerged as a prominent tool for genome editing across various cyanobacterial species [2, 40]. To enable precise control over gene expression, the nuclease activity of Cas12a was rendered inactive by introducing a single mutation, D917A, resulting in a catalytically dead Cas12a (dCas12a) that retains its DNA binding capability. This modification allows for the steric repression of transcription, facilitating effective gene regulation. In fast-growing cyanobacterium *Synechococcus elongatus* UTEX 2973, dCas12a can be expressed constitutively from a strong promoter [24], contrasting with the tight regulation required for dCas9 expression in *Synechococcus* sp. PCC 7002 to achieve transformants [17]. This disparity suggests that dCas12a exerts considerably lower toxicity compared to dCas9 in cyanobacterial cells. However, high levels of dCas12a expression have been associated with some adverse effects, such as reduced photosystem I and chlorophyll content [24]. Therefore, in an effort to mitigate potential detrimental effects associated with dCas12a expression, we opted for the rhamnose-inducible promoter for the transcriptional regulation of both dCas12a and CRISPR RNA (crRNA), previously demonstrated as a superior inducible system in *Synechocystis* for reliable gene expression control [21]. This system relies on the constitutive expression, mediated by the J23119 promoter, of *rhaS* encoding a positive transcriptional regulator to activate transcription at the P*rha* promoter in response to rhamnose (Fig. 1D).

The construction of the pBB_dCas12a vector, harboring the dCas12a and crRNA expression cassettes under the control of the rhamnose-inducible P*rha* promoter, was achieved utilizing a broad-host-range self-replicating plasmid carrying the RSF1010 replication genes (Fig. 1D). With the established CRISPRi-dCas12a system, induction with rhamnose triggers the expression of dCas12a, forming a complex with crRNA to initiate target-specific gene repression.

**Fig. 2:**
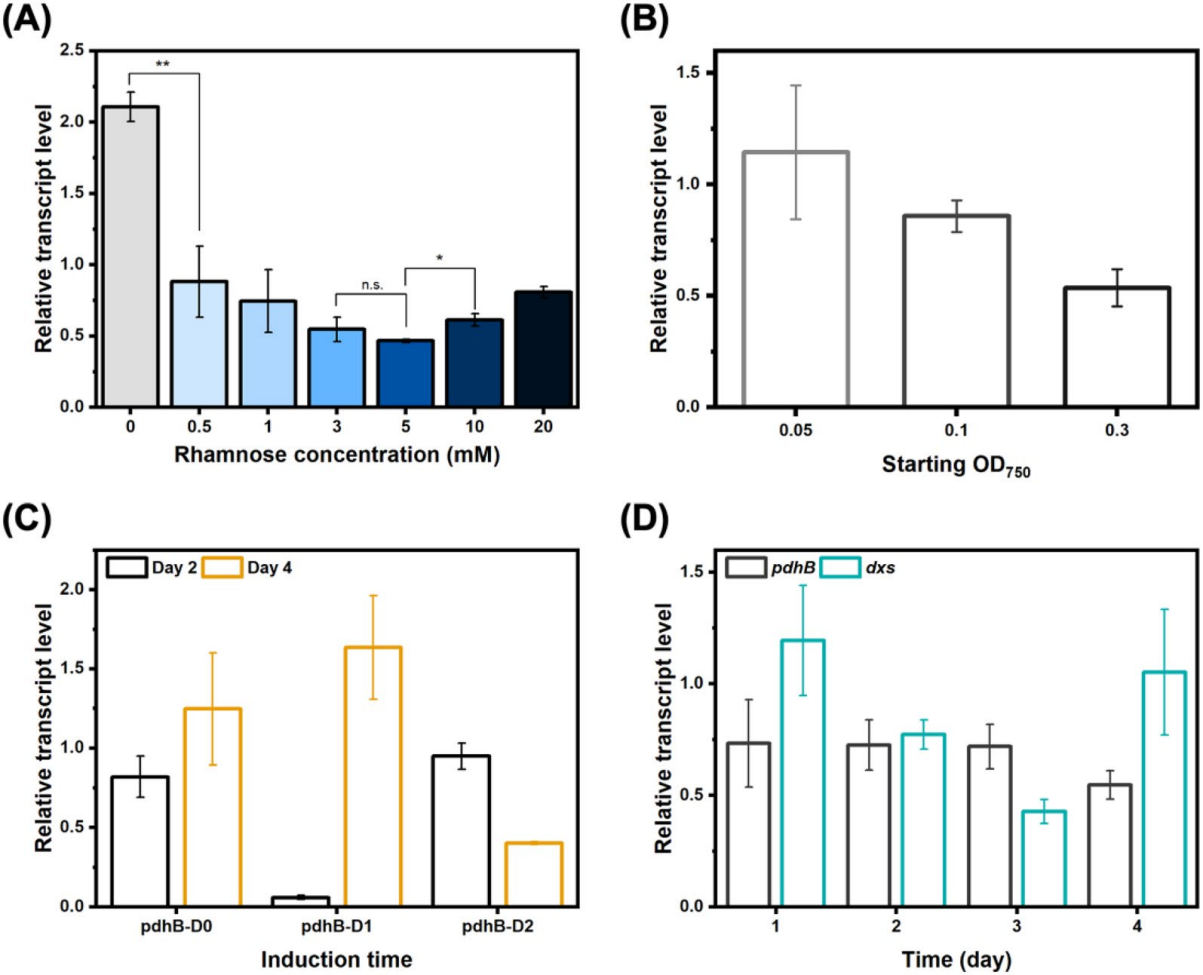
Target downregulation using the rhamnose-inducible CRISPRi-dCas12a tool in *Synechocystis*. **A** *pdhB* downregulation on day 2 with different rhamnose concentrations added on day 0. **B** *pdhB* downregulation on day 2 with 3 mM rhamnose added on day 0 in cultures with initial OD_750_ of 0.05, 0.1, or 0.3. **C** *pdhB* downregulation on days 2 and 4 with 3 mM rhamnose added at different timepoint. pdhB-D0: induction on day 0 during culture inoculation. pdhB-D1: induction on day 1. pdhB-D2: induction on day 2. **D** *pdhB* and *dxs* downregulation on days 1, 2, 3, and 4 with 3 mM rhamnose added on day 0. Relative expression levels were calculated by comparing targeted strains to the control strain BB3 undergoing identical treatments. Results are the mean of three biological replicates. Error bars represent standard deviation. Asterisk represents significant difference between strains (t-Test, *p < 0.05, **p < 0.005)

To initiate tool characterisation, we first targeted the essential gene *pdhB* (*sll1721*), which encodes the pyruvate dehydrogenase subunit β. This step aimed to evaluate and optimise tool efficacy. The optimal rhamnose concentration needed for target downregulation was first tested, using concentrations ranging from 0.5 to 20 mM, expected to induce a moderate expression level according to previous promoter characterisation [3]. Strong target downregulation (Fig. 2A), up to 50%, was achieved with 3–5 mM rhamnose, establishing this CRISPRi tool as efficient for downregulation of essential genes in *Synechocystis*. Lower rhamnose concentrations were presumably insufficient for adequate expression of the CRISPRi elements, preventing target downregulation. Surprisingly, increasing rhamnose concentrations above 5 mM reduced *pdhB* downregulation. The reason for this phenomenon is unclear and requires further attention for elucidation. We tentatively put forward the hypothesis that higher rhamnose concentrations lead to high dCas12 expression, which may be associated with toxicity and increased stress on the cells. In contrast to CRISPRi tools in other organisms [14, 27], this data thus suggested that levels of downregulation and rhamnose concentrations are not linearly correlated for this system. Instead, an optimal inducer concentration window, between 3 and 5 mM, exists for strong target downregulation. Accordingly, subsequent experiments were performed with 3 mM rhamnose for CRISPRi induction, unless otherwise stated.

To further characterise this CRISPRi tool and better understand its behaviour, two additional parameters, starting cell density and induction times, were investigated. *pdhB* downregulation was increased with higher starting cell densities (Fig. 2B). We hypothesised that more biomass provided protection against potential stress and toxicity caused by *pdhB* downregulation. In addition, *pdhB* downregulation was achieved, regardless of the induction times (Fig. 2C, and Fig. S1), albeit with varying efficiency, further highlighting tool flexibility. Importantly, these results suggest that maximal downregulation seemed to be attained 24–48 h post-induction while weaker downregulation was observed on day 4, indicating a potential downregulation reduction at later growth phases. While rhamnose has previously induced strong and stable expression in *Synechocystis* [3, 21], we hypothesised that loss of CRISPRi induction was possibly occurring, leading to the observed reduced downregulation. To overcome this, an additional rhamnose induction on day 2 was investigated to maintain stable target downregulation. However, this strategy did not stabilise downregulation level and even caused erratic tool behaviour when added rhamnose concentration was normalised to cell density (Fig. S2). It remains unclear if weaker downregulation at later growth phases is linked to rhamnose induction or to higher cell density and associated regulatory mechanisms. Since downregulation stabilisation was not further investigated, a consistent reduction of CRISPRi effects was observed at later timepoints across various experiments. As such, this tool may not be appropriate for long-term batch experiments requiring sustained target downregulation but allows rapid screening of competing pathways.

A second target, *dxs* (*sll1945*), encoding for 1-deoxy-d-xylulose 5-phosphate synthase, was downregulated with CRISPRi (Fig. 2D, and Fig. S1) to further confirm tool applicability. While *dxs* downregulation was successfully accomplished, a different repression pattern was observed, with a strong but transient downregulation of *dxs* at 72 h post-induction. This further emphasises target-specific tool behaviour variations, which may partly be caused by variability in gRNA binding and tool targeting, intrinsic to CRISPR-based tools [10, 44]. However, target function will also impact tool behaviour, as exemplified by the distinct behaviour observed for both *pdhB* and *dxs*, involved in acetyl-CoA formation and carotenoid biosynthesis, respectively. Target metabolic importance and regulation can therefore influence CRISPRi performance, suggesting the need for target-specific and/or application-specific tool characterisation for use in *Synechocystis*.

These results validate the utility of the described CRISPRi tool for strong downregulation of targets involved in diverse metabolic pathways, providing a better understanding of tool behaviour, although target-specific variations were observed. We then employed CRISPRi to identify relevant targets for repression for enhancement of isobutanol (IB)/3-methyl-1-butanol (3M1B) production.

### Impacts of dCas12a protein expression on IB/3M1B-producing *Synechocystis* strain

The *Synechocystis* cell demonstrates the capability to produce IB and 3M1B upon heterologous expression of α-ketoisovalerate decarboxylase (Kivd), a key enzyme in the 2-keto acid pathway [31]. Notably, modulation of *kivd*^*S286T*^ copy numbers successfully enhances IB and 3M1B production. In this study, to achieve optimal IB/3M1B production, strain HX11 was engineered with two copies of *kivd*^*S286T*^ integrated into the genome (Fig. 1C), establishing an IB/3M1B-producing base strain to evaluate the CRISPRi-dCas12a system. The first *kivd*^*S286T*^ copy was integrated into the *ddh* locus, while the second copy was integrated into the *slr0168* locus. Both copies were placed under the control of the P*trc*BCD promoter to ensure constitutive and strong expression. Validation of protein expression was conducted through Western blot analysis (Fig. S3 A).

To evaluate the impact of the dCas12a protein on IB/3M1B production, two strains, HX11-EVC and HX11-pEEK*, were engineered by introducing pBB_dCas12a and pHX22 plasmids into strain HX11, respectively (Fig. 1D, Fig. S3B, Table 1, and Table S1). Strain HX11-EVC, expressing dCas12a, exhibited a similar growth rate and IB/3M1B production per cell compared to the control strain HX11-pEEK* (Fig. S4). Therefore, we conclude that under the regulation of the rhamnose-inducible promoter, the expressed dCas12 protein exhibited minimal impacts on cell growth and IB/3M1B production, validating that the CRISPRi-dCas12a system was suitable to explore diverse gene targets for repression to enhance IB/3M1B production. These findings are consistent with previous studies indicating low toxicity of the dCas12a protein in cyanobacteria [40].

### Targets chosen for repression using CRISPRi-dCas12a system

Extending from the central carbon metabolite pyruvate, the 2-keto acid pathway for IB/3M1B production encounters competition from numerous pathways that may divert carbon fluxes. With the development of the CRISPRi-dCas12a system, there is an opportunity to broaden the spectrum of rewiring native metabolic networks, particularly targeting essential genes where deletions prove detrimental to cell viability. To redirect carbon flux towards the 2-keto acid pathway, we designed crRNAs targeting fifteen candidate genes encoding enzymes involved in core metabolic pathways potentially limiting available precursors for IB/3M1B production (Fig. 1E). Sucrose phosphate synthase (Sps) and fructose 1,6-bisphosphatase (Fbp) were chosen due to their involvement in sucrose metabolism, diverting carbon flux away from pyruvate. Key enzymes including phosphoenolpyruvate (PEP) carboxylase (encoded by *ppc*), citrate synthase (encoded by *gltA*), aconitase (AcnB), isocitrate dehydrogenase (encoded by *icd*), and fumarase (FumC) from the tricarboxylic acid cycle (TCA cycle) were selected to enhance the pyruvate pool. Additionally, pyruvate dehydrogenase (Pdh), phosphoenolpyruvate synthase (encoded by *ppsA*), and 3-deoxy-7-phosphoheptulonate synthase (CcmA) were targeted to further increase the pyruvate pool. Branched-chain amino acid aminotransferase (IlvE) was targeted to reduce carbon utilization in branched-chain amino acid biosynthesis. Phycocyanin β-subunit (CpcB) was selected to improve photosynthesis efficiency and culture productivity by minimizing the phycobilisome light-harvesting antenna size [22]. Lastly, we also selected acetyl-CoA acetyltransferase (PhaA), acetyl-CoA carboxylase (AccC), acetyl-CoA hydrolase (Ach), and phosphotransacetylase (Pta) for downregulation, as they are essential for key steps in poly-3-hydroxybutyrate (PHB), fatty acids, and acetate biosynthesis, respectively, thus diverting carbon into these products.

### lmplementation and validation of the CRISPRi-dCas12a system in the IB/3M1B-producing *Synechocystis* strain

**Fig. 3:**
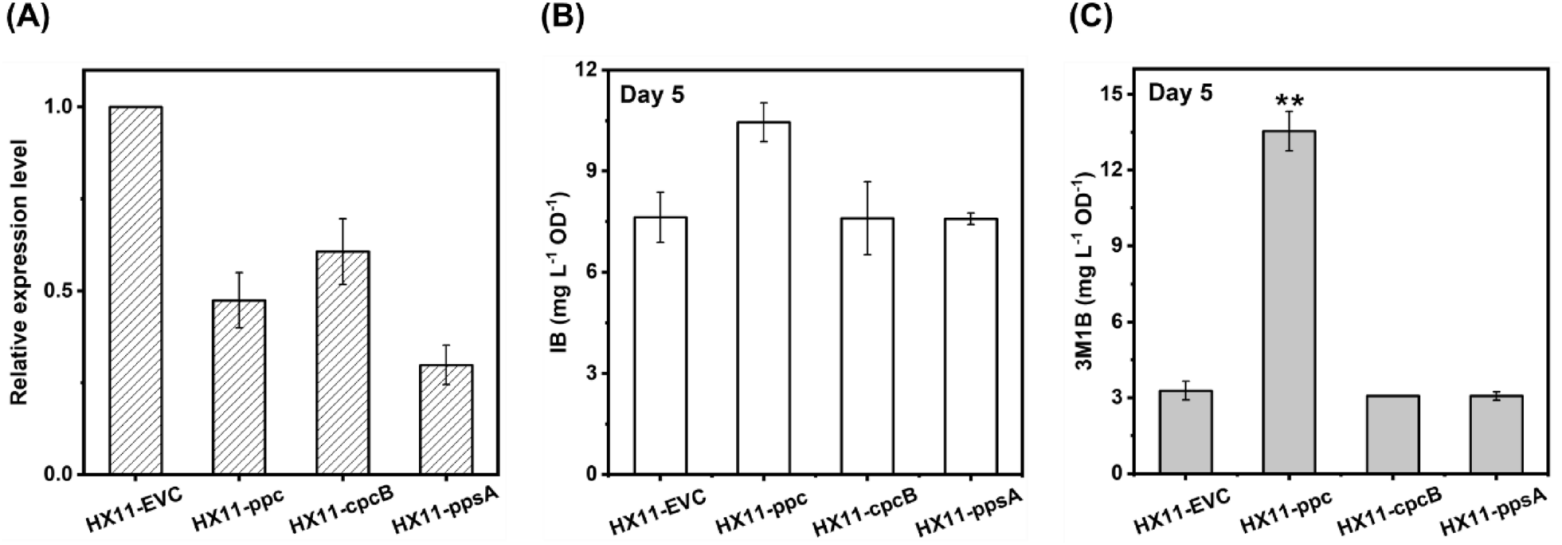
Establishment of dCas12a-mediated CRISPRi system in isobutanol (IB)/3-methyl-1-butanol (3M1B)-producing *Synechocystis* strain HX11. **A** Relative mRNA expression level of *ppc*, *cpcB*, and *ppsA* genes in HX11-ppc, HX11-cpcB, and HX11-ppsA strains, respectively, compared to the level of HX11-EVC strain. **B** IB production per cell of each strain with selected gene repressed on Day 5. **C** 3M1B production per cell of each strain with selected gene repressed on Day 5. For IB/3M1B production, results are the mean of two biological replicates; for mRNA relative expression level, results are the mean of three technical replicates. Error bars represent standard deviation. Asterisk represents significant difference between different strains (t-Test, *p < 0.05, **p < 0.005)

Before systematically assessing the impact of repressing the proposed fifteen gene targets on IB/3M1B production, we selected *ppc*, *cpcB*, and *ppsA* as priority targets for repression. This choice aimed to validate the functionality of the developed CRISPRi-dCas12a system in the IB/3M1B-producing *Synechocystis* strain HX11. Three recombinant strains (HX11-ppc, HX11-cpcB, and HX11-ppsA) were generated by introducing the pBB_dCas12a-derived plasmids, containing target-specific crRNA, into strain HX11 (Table 1, and Table S1). Simultaneously, a control strain, HX11-EVC, was constructed by introducing a pBB_dCas12a (without target-specific crRNA) into strain HX11 (Table 1, and Table S1). As expected, the expression levels of *ppc*, *cpcB*, and *ppsA* in strains HX11-ppc, HX11-cpcB, and HX11-ppsA, respectively, were significantly reduced compared to in the control strain HX11-EVC (Fig. 3A).

Despite the observed repression of the target essential genes, there was no significant growth rate difference among the tested strains (Fig. S3 C). As illustrated in Fig. 3B, C, inhibition of *cpcB* and *ppsA* did not have positive effects on IB/3M1B production per cell on day 5. However, compared to control strain, 3M1B production was markedly enhanced in strain HX11-ppc through inhibition of the *ppc* gene expression, while achieving similar IB production per cell (Fig. 3B, C). This demonstrates the successful implementation of the CRISPRi-dCas12a system in conjunction with the heterologous 2-keto acid pathway in *Synechocystis* for IB and 3M1B production. Furthermore, the favorable effects on 3M1B production resulting from *ppc* inhibition suggest that the CRISPRi-dCas12a system holds promise as an approach for systematic screening of comprehensive competing pathways for IB/3M1B production.

### Condition optimization for application of CRISPRi-dCas12a system in the IB/3M1B-producing *Synechocystis* strain

The effective application of CRISPRi-dCas12a system in the IB/3M1B-producing *Synechocystis* strain necessitates careful optimization of cultivation conditions. Such optimization ensures the robust functionality and reliable performance of the CRISPRi-dCas12a system within the specific cellular context of the engineered *Synechocystis* strain. In this section, we systematically analyzed three parameters: rhamnose induction timepoint; rhamnose concentration for induction; and culture with HCl titration.

#### Rhamnose induction timepoint

The rhamnose-inducible promoter P*rha* was employed to regulate the expression of dCas12a and crRNA to investigate whether their expression levels could be controlled during different growth stages of *Synechocystis* strains through varying timepoints for rhamnose addition. Two strains, HX11-EVC and HX11-ppc, were selected to evaluate the impact of 3 mM rhamnose addition at five specific timepoints: before inoculation, day 0, day 1, day 2, and day 3, with a control group without rhamnose addition included for comparison.

**Fig. 4:**
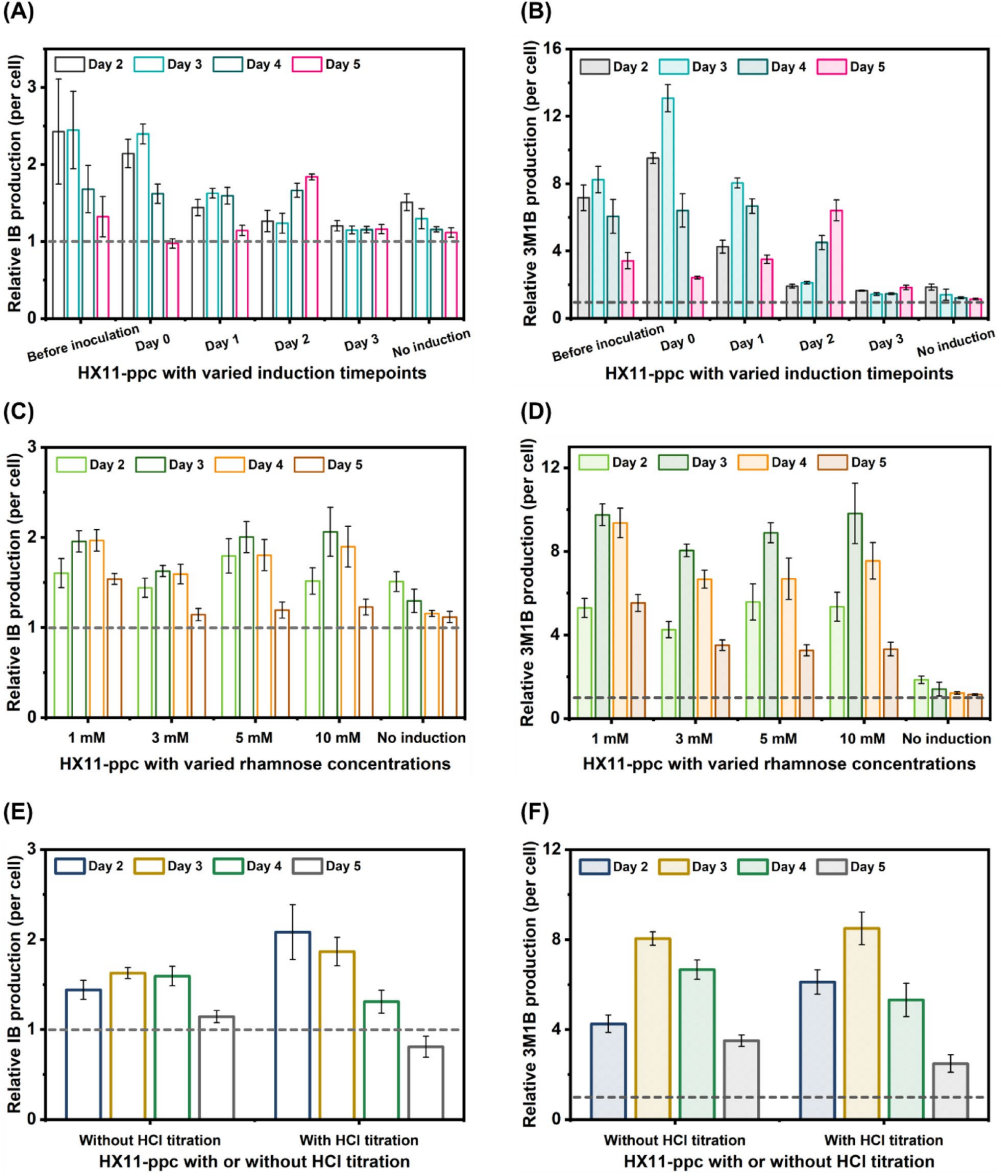
Test of dCas12a-mediated CRISPRi system in isobutanol (IB)/3-methyl-1-butanol (3M1B)-producing *Synechocystis* strain with three different conditions: rhamnose induction timepoints, rhamnose concentrations for induction, and HCl titration. **A** Relative IB production per cell of strain HX11-ppc with varied rhamnose induction timepoints on days 2, 3, 4, and 5, compared to the levels of strain HX11-EVC. Three millimolar rhamnose were added for induction in strains HX11-EVC and HX11-ppc before inoculation, on days 0, 1, 2, 3, or no induction. **B** Relative 3M1B production per cell of strain HX11-ppc with varied rhamnose induction timepoints on days 2, 3, 4, and 5, compared to the levels of strain HX11-EVC. **C** Relative IB production per cell of strain HX11-ppc with varied rhamnose concentrations for induction on days 2, 3, 4, and 5, compared to the levels of strain HX11-EVC. Rhamnose (0 mM, 1 mM, 3 mM, 5 mM, or 10 mM) was added for induction in strains HX11-EVC and HX11-ppc on day 1. **D** Relative 3M1B production per cell of strain HX11-ppc with varied rhamnose concentrations for induction on days 2, 3, 4, and 5, compared to the levels of strain HX11-EVC. **E** Relative IB production per cell of strain HX11-ppc cultivated with or without HCl titration on days 2, 3, 4, and 5, compared to the levels of strain HX11-EVC. Three millimolar rhamnose were added for induction in strains HX11-EVC and HX11-ppc on day 1. The target pH range of the cultures with HCl titration was between 7 and 8. **F** Relative 3M1B production per cell of strain HX11-ppc cultivated with or without HCl titration on days 2, 3, 4, and 5, compared to the levels of strain HX11-EVC. Results are the mean of three biological replicates. Error bars represent standard deviation

While strain HX11-EVC exhibited consistent growth patterns and IB/3M1B production per cell across all tested timepoints for rhamnose addition (Fig. S5 A-C), strain HX11-ppc showed notable differences in growth patterns and IB/3M1B production per cell with varying timepoints of rhamnose addition (Fig. S5D-F). Earlier rhamnose induction for an active CRISPRi-dCas12a system in strain HX11-ppc led to a prolonged growth lag-stage (Fig. S5D). Interestingly, no significant differences were observed in cell growth or IB/3M1B production per cell between group with 3 mM rhamnose added on day 3 and that without rhamnose addition (Fig. S5D-F). Compared to strain HX11-EVC under all tested conditions, strain HX11-ppc with 3 mM rhamnose added on day 0 exhibited higher relative IB/3M1B production per cell (Fig. 4A, B).

Specifically, when rhamnose was added to the HX11-ppc strain before inoculation, on day 0 and day 1, a lower OD_750_ but higher IB/3M1B production per cell were achieved on day 2 (Fig. S5D-F), suggesting a redirection of carbon flux from biomass accumulation to IB/3M1B biosynthesis. Notably, relative IB/3M1B production per cell was significantly increased in strain HX11-ppc without rhamnose addition (HX11-ppc_0) compared to strain HX11-EVC without rhamnose addition (HX11-EVC_0), especially on day 2 and day 3 (Fig. 4A, B). This observation indicates minimal expression of dCas12a and crRNA at the P*rha* promoter without rhamnose induction. Overall, rhamnose addition on day 0 is preferred for CRISPRi-dCas12a system to achieve optimal gene repression and IB/3M1B production in *Synechocystis*.

#### Rhamnose concentration for induction

As demonstrated in “Initial design and characterization of CRISPRi-dCas12a system in* Synechocystis*” section, the optimal window for achieving strong repression of *pdhB* with rhamnose addition falls within the range of 3–5 mM. Expanding upon this finding, to determine the ideal rhamnose concentration tailored specifically for the CRISPRi-dCas12a system in IB/3M1B-producing *Synechocystis* strain, five different rhamnose concentrations (0 mM, 1 mM, 3 mM, 5 mM, or 10 mM) were tested on strain HX11-ppc. The control strain HX11-EVC was included for comparison. Similar growth patterns and IB/3M1B production per cell were observed for strain HX11-EVC across all rhamnose concentrations ranging from 0 to 10 mM (Fig. S6 A-C). Likewise, strain HX11-ppc exhibited consistent growth patterns and IB/3M1B production per cell within rhamnose concentration range between 1 and 10 mM (Fig. S6D-F). Notably, in the absence of rhamnose addition, HX11-ppc displayed enhanced growth during the exponential phase, reaching maximal OD_750_ two days earlier than with 1–10 mM rhamnose addition (Fig. S6D). Additionally, HX11-ppc without rhamnose addition exhibited significantly lower IB/3M1B production per cell (Fig. S6E-F). Interestingly, compared to HX11-EVC strain across all tested rhamnose concentrations, strain HX11-ppc with 1–10 mM rhamnose added demonstrated comparable relative IB and 3M1B production per cell (Fig. 4C, D). Therefore, rhamnose concentrations within the range of 1–10 mM, consistent with earlier results (Fig. 2A), proved effective in inducing the CRISPRi-dCas12a system for target gene repression in IB/3M1B-producing *Synechocystis* strain.

#### Culture with HCl titration

Cultivating *Synechocystis* under a controlled pH via HCl titration has been identified as advantageous for both cell growth and IB/3M1B production [32]. Consistently, HX11-EVC demonstrated increased maximal OD_750_ and IB/3M1B production per cell when maintained at a controlled pH between 7 and 8 through daily HCl titrations. Under the HCl titration regime, HX11-ppc showed enhanced IB and 3M1B production per cell on days 2–4 and days 2–3, respectively (Fig. S7B-C). Furthermore, as illustrated in Fig. 4E, F, in comparison to HX11-EVC strain with or without HCl titration, strain HX11-ppc with HCl titration exhibited higher relative IB/3M1B production per cell on days 2–3 but lower relative IB/3M1B production per cell on days 4–5.

The influence of rhamnose induction timing, rhamnose concentration, and HCl titration on the CRISPRi-dCas12a system in IB/3M1B-producing *Synechocystis* was systematically investigated. There were no discernible differences in relative IB/3M1B production per cell across various tested rhamnose concentrations in strain HX11-ppc (Fig. 4C, D). Similarly, pH-adjusted cultures had minimal impacts on relative IB/3M1B production per cell (Fig. 4E, F). Notably, rhamnose induction on day 0 resulted in significantly higher relative 3M1B production per cell (Fig. 4B). These optimized conditions serve as the basis for successful implementation of the CRISPRi-dCas12a system in IB/3M1B-producing *Synechocystis* strain, facilitating precise modulation of gene expression to enhance IB/3M1B production. It is important to note that these tests were conducted with the *ppc* target, different target genes may exhibit varied transcription patterns under different environmental conditions [34]. Therefore, the determined parameter may not be optimal for all gene targets. Despite this, due to constraints in time and resources, the determined optimal condition was employed to map potential competing pathways using the CRISPRi-dCas12a system. Specifically, IB/3M1B-producing *Synechocystis* strain integrated with CRISPRi-dCas12a system will be cultured without HCl titration, with 3 mM rhamnose added for CRISPRi-dCas12a system activation on day 0. For future endeavors, it would be beneficial to reassess the abovementioned parameters for individual targets that exhibit positive effects on IB/3M1B enhancement, to optimize the IB/3M1B production capacity.

### Systematic application of CRISPRi-dCas12a for IB/3M1B production in *Synechocystis*

**Fig. 5:**
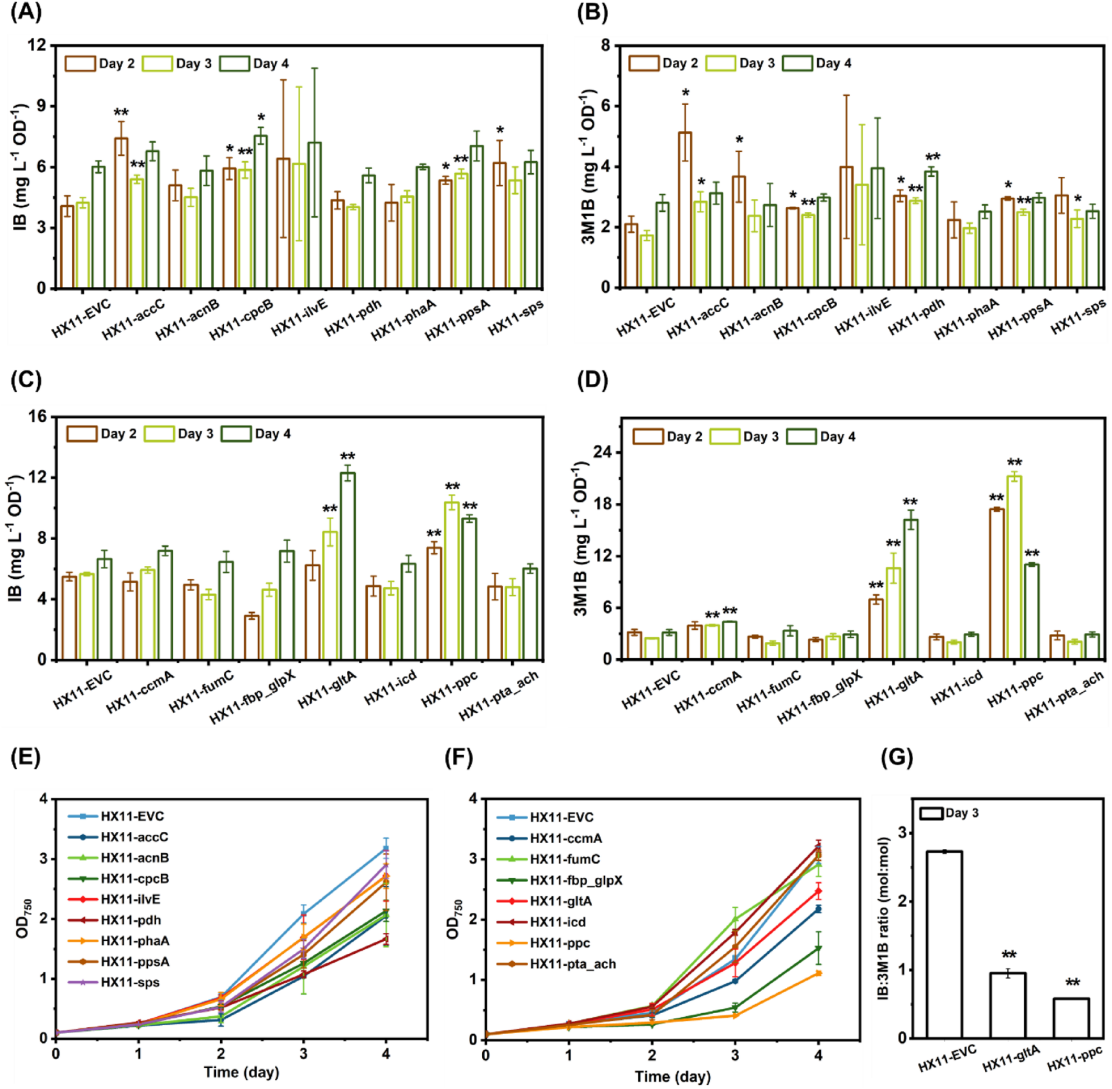
Application of dCas12a-mediated CRISPRi system for selected target gene(s) repression in isobutanol (IB)/3-methyl-1-butanol (3M1B)-producing *Synechocystis* strain. See Table 1 for the details of the strains. Three millimolar rhamnose were added on day 0 for induction in individual strain. **A** IB production per cell of the engineered *Synechocystis* strains with selected gene(s) repressed on days 2, 3, and 4 (first batch). **B** 3M1B production per cell of the engineered *Synechocystis* strains with selected gene(s) repressed on days 2, 3, and 4 (first batch). **C** IB production per cell of the engineered *Synechocystis* strains with selected gene(s) repressed on days 2, 3, and 4 (second batch). **D** 3M1B production per cell of the engineered *Synechocystis* strains with selected gene(s) repressed on days 2, 3, and 4 (second batch). **E** Growth profile of the engineered *Synechocystis* strains with selected gene(s) repressed (first batch). **F** Growth profile of the engineered *Synechocystis* strains with selected gene(s) repressed (second batch) **G** Molar ratio of IB and 3M1B in engineered *Synechocystis* strains HX11-EVC, HX11-gltA, and HX11-ppc on day 3. Results are the mean of three biological replicates. Error bars represent standard deviation. Asterisk represents significant difference between engineered strains and the control strain (t-Test, *p < 0.05, **p < 0.005)

During strain development, the homologous recombination approach has been extensively employed for target gene knock-in or knock-out in metabolic engineering of cyanobacteria for bioproduction. However, due to the polyploidy nature of *Synechocystis*, generating gene(s) knock-out can be time-consuming. Moreover, in previous studies utilizing *Synechocystis* cells for IB/3M1B production [45, 47], it was challenging to completely knock out genes essential for cell viability using the traditional homologous recombination approach, making it difficult to identify potential competing pathways. The newly developed CRISPRi-dCas12a system offers an ideal solution for rapidly evaluating the effects of gene downregulation on IB/3M1B production, particularly for those essential for cell growth. Fifteen pBB_dCas12a-derived plasmids containing target(s)-specific crRNAs were constructed and introduced into strain HX11 to generate fifteen strains with individual gene(s) targeted for repression (Table 1, and Table S1). Plasmid pBB_dCas12a was also introduced into strain HX11 to generate a control strain, designated HX11-EVC. Due to shaker capacity limitations, the engineered strains were divided into two separate batches, with control strain HX11-EVC included in each batch for comparison. Out of the fifteen proposed gene targets for repression, more than half (ten targets) exhibited significantly positive effects on IB and/or 3M1B production (Fig. 5A–D). However, since many of the selected targets are essential for cell viability, partial repression resulted in significant cell growth retardation (Fig. 5E, F). To address this, the observed severe growth inhibition caused by dCas12a-mediated essential genes repression could potentially be mitigated by designing crRNAs targeting further downstream of the coding regions to achieve lower repression efficiency [19]. Meanwhile, with lower repression efficiency, the resulting IB/3M1B production enhancement may decrease accordingly.

Single repression of *ppc* resulted in the least cell growth (Fig. 5F), highlighting its essential role, likely due to its critical involvement in CO_2_ assimilation and as a crucial link between central carbon metabolism and the TCA cycle. This observed growth inhibition aligns with a similar study by Cheah et al. [8]. Overexpression of PEP carboxykinase (encoded by *pck*), as a reverse reaction of *ppc* catalyzed enzymatic reaction in *Synechococcus*, resulted in dramatically reduced growth rate [8]. Single repression of *ppc* in strain HX11-ppc led to a 1.8-fold increase in IB production per cell and an 8.6-fold increase in 3M1B production per cell on day 3 (Fig. 5C, D). Additionally, substantial redistribution of IB/3M1B products was observed in response to *ppc* repression (Fig. 5G). However, due to the reduced growth rate caused by *ppc* repression, strain HX11-ppc achieved a lower IB titer and a slightly higher 3M1B titer compared to strain HX11-EVC (Fig. S8).

*gltA*, chosen as the second target from the TCA cycle for repression, was analyzed for effects on cell growth and IB/3M1B production per cell (Fig. 1E). Although there was no significant difference in OD_750_ between the strain with *gltA* repression (HX11-gltA) and the strain without *gltA* repression (HX11-EVC) on days 1–3, a significant decrease in OD_750_ was observed on day 4 for the strain with *gltA* repression (Fig. 5F). Specifically, the OD_750_ values for strains HX-gltA and HX-EVC were 2.5 and 3.1, respectively (Fig. 5F). On day 4, a 2.1-fold and a 5.7-fold improvement in IB and 3M1B production per cell, respectively, was observed after *gltA* repression (Fig. 5C, D). Furthermore, IB/3M1B redistribution was observed, albeit to a lesser extent compared to *ppc* repression (Fig. 5G).

With repression of *ppc* and *gltA*, there was a notable increase in overall IB/3M1B production per cell (Fig. 5C, D), likely stemming from the rerouting of the fixed carbon towards the heterologous 2-keto acid pathway for IB/3M1B biosynthesis, departing from the TCA cycle. The unexpected products redistribution of IB and 3M1B (Fig. 5G) warrants further investigation, potentially linked to alterations in pyruvate and amino acid metabolism [8]. As one hypothesis, *ppc*/*gltA* repression may lead to modified valine and leucine biosynthesis, favoring leucine over valine, consequently resulting in the dramatically increased 3M1B production after heterologous expression of Kivd. Firstly, *ppc*/*gltA* repression may directly or indirectly enhance flux through the leucine biosynthesis pathway by regulating the *leuABCD* expression level. Secondly, *leuA* encoded 2-isopropylmalate synthase uses 2-ketoisovalerate, acetyl-CoA, and water for 2-isopropylmalate synthesis. In strains with *ppc*/*gltA* repression, less acetyl-CoA is allocated for citrate synthesis, implying that more acetyl-CoA may be available for 2-isopropylmalate synthase-catalyzed reactions. As a result, there will be more precursor available for 3M1B biosynthesis. However, elucidating the underlying mechanisms requires additional exploration.

Three additional targets from the TCA cycle, namely *acnB*, *icd*, and *fumC*, were selected for downregulation. Repression of *icd* and *fumC* did not yield any positive effects on IB/3M1B production (Fig. 5C, D). However, *acnB* repression exhibited a slight increase in 3M1B production per cell on day 2 (Fig. 5B), with no discernible effects on IB production from day 2 to day 4 (Fig. 5A). The observed positive effects of *acnB* repression may be attributed to the enlarged pyruvate pool, as *acnB* repression reduces cellular biomass accumulation [9]. Moreover, *acnB* repression led to citrate accumulation in the cells, and changes induced by citrate in the pentose phosphate pathway might contribute to alterations in metabolite levels within the 2-keto acid pathway [11].

In addition to *ppc*, three more genes (*ccmA*, *ppsA*, and *pdh*) were targeted for repression to potentially enhance the pyruvate pool, a crucial precursor for IB/3M1B biosynthesis. As shown in Fig. 5A–D, repression of these three genes individually resulted in significantly enhanced IB and/or 3M1B production. *ccmA* encodes DAHP synthase in *Synechocystis*, initiating the shikimate pathway by condensing phosphoenolpyruvate (PEP) and erythrose-4-phosphate (E4P) to form 3-deoxy-D-arabino-heptulosonate-7-phosphate (DAHP) [7]. *ppsA* encodes PEP synthase, catalyzing a reaction using pyruvate as a substrate for PEP biosynthesis. Overexpression of *ppsA* has been shown to enhance ethylene production by increased carbon flux towards PEP and further flow into the TCA cycle [12]. Hence, repressing *ppsA* may redirect carbon away from the TCA cycle. *pdh*-encoded pyruvate dehydrogenase is responsible for converting pyruvate into acetyl-CoA, which enters the TCA cycle or is utilized for other chemical production. Partial inhibition of *pdh* could effectively block carbon flux towards acetyl-CoA, augmenting pyruvate-derived IB/3M1B production (Fig. 5A, B). Our findings are consistent with a recent report indicating that *pdh* knockdown using antisense RNA elevates pyruvate partition and aldehyde productivity in *Synechococcus* [8].

To evaluate if downregulating the sucrose synthesis pathway contributes to improved IB/3M1B production, *fbp* (encoded by *slr0952* and *slr2094* in *Synechocystis*) and *sps* were selected as targets for repression. While *fbp* repression showed no positive effects (Fig. 5C, D), *sps* repression resulted in a 1.5-fold improvement on IB production per cell on day 2 and a 1.3-fold improvement on 3M1B production per cell on day 3 (Fig. 5A, B).

As a commonly employed strategy to optimize photosynthesis efficiency and culture productivity by reducing the phycobilisome light-harvesting antenna size [22], *cpcB* was targeted for repression. This approach aimed to downregulate the expression of the phycocyanin β-subunit, a pivotal component of the phycobilisome light-harvesting antenna (Fig. 1E). As anticipated, both IB and 3M1B production per cell showed significant improvements (Fig. 5A, B). Previous studies have shown that strains with truncated light-harvesting antenna (TLA) exhibit enhanced photosynthesis efficiency and biomass productivity under simulated bright sunlight and high cell-density conditions [22]. However, in our current investigation, strain HX11-cpcB, which harbors a truncated light-harvesting complex, was cultivated under 50 μmol photons m^−2^ s^−1^. To further optimize growth and potentially increase IB/3M1B production per cell, higher light intensities may be preferred.

PHB, fatty acids, and acetate biosynthesis pathways represent potential competing pathways with the 2-keto acid pathway for IB/3M1B production. Repression of *phaA*, *ach* and *pta* did not improve IB or 3M1B production per cell in strains HX11-phaA and HX11-pta_ach (Fig. 5A–D). Conversely, strain HX11-accC exhibited higher IB/3M1B production per cell on days 2–3 (Fig. 5A, B) compared to the control strain (HX11-EVC), indicating that *accC* repression effectively redirects carbon flux from fatty acids biosynthesis to IB/3M1B biosynthesis.

Lastly, considering that the 2-keto acid pathway shares precursors with valine and leucine biosynthesis, *ilvE* was targeted for repression to shift valine/leucine biosynthesis towards IB/3M1B biosynthesis. Significant variations were observed on growth and IB/3M1B production per cell among the three tested independent cell lines (Fig. 5A, B, and E), making it challenging to determine its positive effects on IB/3M1B production. Therefore, further characterization was performed on the three cell lines (HX11-ilvE1, 2, and 3) with technical triplicates (Fig. S9). Lower OD_750_ values were achieved by all three cell lines, compared to the control strain. Consistently, significant variations in IB/3M1B production per cell were observed among the three cell lines (Fig. S9B-C). Significant improvement was obtained for 3M1B production per cell on days 2–4 and for IB production per cell on day 2, after combining data from the three cell lines with *ilvE* repression (Fig. S9D-E). The observed variations with *ilvE* repression may be attributed to its essential role in branched-chain amino acid biosynthesis, which are crucial for growth, metabolism, and stress response of *Synechocystis* [23]. In conclusion, *ilvE* repression has positive effects on IB/3M1B production per cell, despite significant variations among biological triplicates.

Out of the fifteen proposed targets, repression of ten resulted in enhanced IB/3M1B production per cell (Fig. 5). Maximal downregulation seemed to be attained 24 to 48 h post-induction for the repression of *pdhB* gene, as demonstrated previously. Based on the IB/3M1B production per cell of strain HX11-ppc (Fig. 5C, D, and Fig. S10), both IB and 3M1B production per cell increased from day 2 to day 3, but started decreasing from day 3, strongly indicating that *ppc* repression was more efficient up to 72 h post-induction. In contrast, both IB and 3M1B production per cell of strain HX11-gltA increased from day 2 to day 5 (Fig. 5C, D, and Fig. S10), suggesting the effective *gltA* repression extended to 120 h post-induction. Hence, the efficacy of the CRISPRi-dCas12a system is target-dependent. Further verification of these observations may involve designing multiple crRNAs for a single gene target. In conclusion, the developed CRISPRi-dCas12a system effectively maps potential competing pathways for IB/3M1B production in *Synechocystis*. Additionally, the identified positive targets for repression may benefit other pyruvate-derived chemical production.

### Efficient multiplex gene repression by CRISPRi-dCas12a using a single crRNA array for enhanced IB/3M1B production in *Synechocystis*

**Fig. 6:**
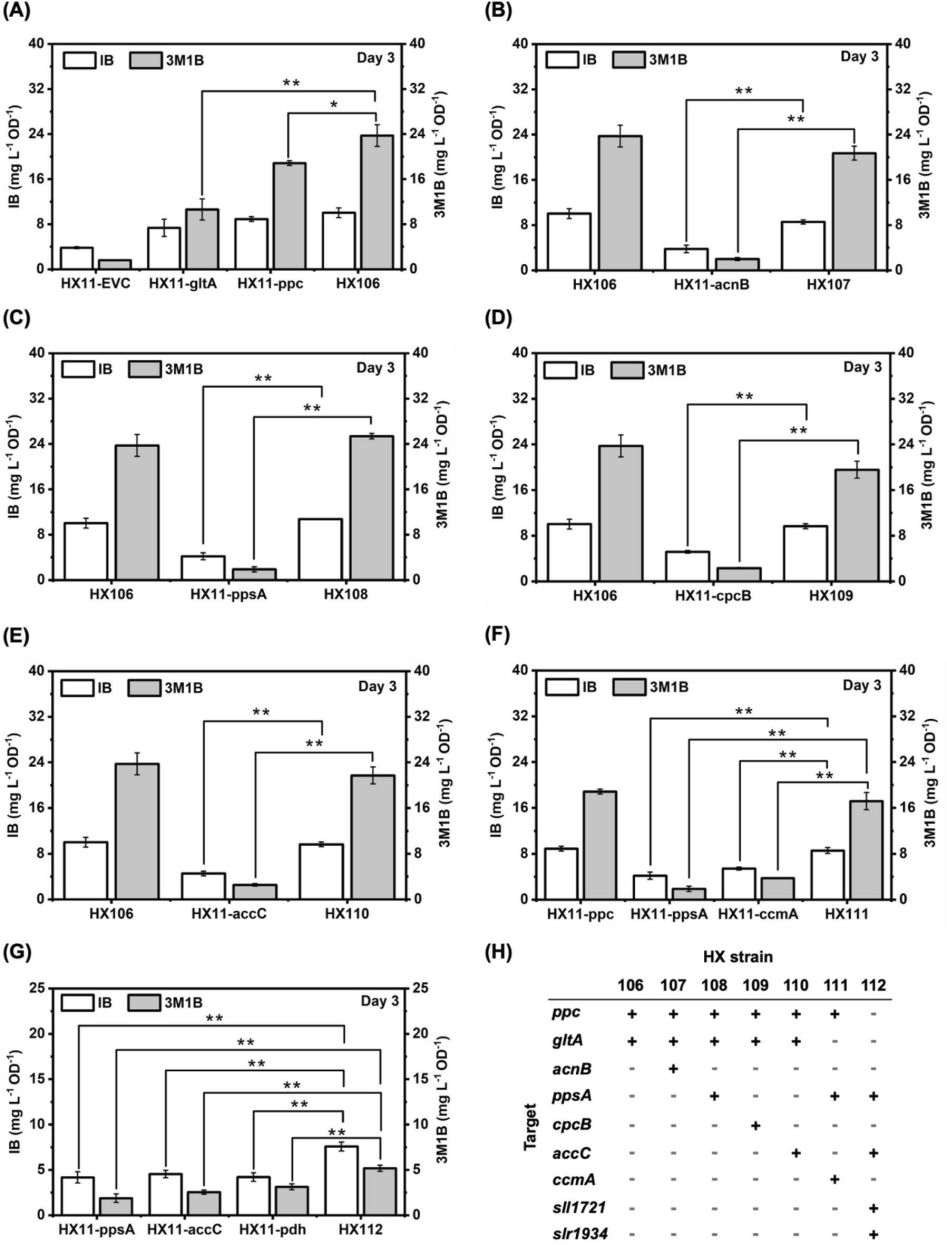
Combinatorial multiplex repression using dCas12a-mediated CRISPRi system for isobutanol (IB)/3-methyl-1-butanol (3M1B) production in *Synechocystis*. See Table 1 for the details of the strains. Three millimolar rhamnose were added on day 0 for induction in individual strain. **A** IB and 3M1B production per cell of the engineered *Synechocystis* strains HX11-EVC, HX11-gltA, HX11-ppc, and HX106 on day 3. **B** IB and 3M1B production per cell of the engineered *Synechocystis* strains HX106, HX11-acnB, and HX107 on day 3. **C** IB and 3M1B production per cell of the engineered *Synechocystis* strains HX106, HX11-ppsA, and HX108 on day 3. **D** IB and 3M1B production per cell of the engineered *Synechocystis* strains HX106, HX11-cpcB, and HX109 on day 3. **E** IB and 3M1B production per cell of the engineered *Synechocystis* strains HX106, HX11-accC, and HX110 on day 3. **F** IB and 3M1B production per cell of the engineered *Synechocystis* strains HX11-ppc, HX11-ppsA, HX11-ccmA, and HX111 on day 3. **G** IB and 3M1B production per cell of the engineered *Synechocystis* strains HX11-ppsA, HX11-accC, HX11-pdh, and HX112 on day 3. **H** Summary of the strains and their respective targets included in this experiment for repression with the CRISPRi system. Results are the mean of three biological replicates. Error bars represent standard deviation. Asterisk represents significant difference between recombinant strains and the control strain (t-Test, *p < 0.05, **p < 0.005)

The CRISPRi-dCas12a system has demonstrated its capacity for multiplex gene repression, enabling simultaneous targeting of up to four genes within a single array [49]. Previous studies have showcased its effectiveness, revealing consistent repression of all four targeted genes with similar fold-changes to those achieved with individual crRNAs [49]. Recently, this system was successfully employed in *Synechococcus* to simultaneously repress three target genes [9]. Therefore, the logical progression was to explore the repression of multiple genes (Fig. 6H) in a single array using the CRISPRi-dCas12a system and assess its impacts on IB/3M1B production per cell in the base strain HX11.

Given that single repression of *ppc* and *gltA* proved most effective in enhancing IB/3M1B production per cell in strain HX11, this combination was pursued. A new strain, designated HX106, with concurrent repression of *ppc* and *gltA* was generated (Table 1). Strain HX106 exhibited notable improvements in both IB and 3M1B production per cell compared to the control strain HX11-EVC (Fig. 6A). Particularly noteworthy was the significant enhancement in 3M1B production per cell in strain HX106 compared to strains with either *ppc* or *gltA* repression alone (Fig. 6A). Interestingly, strain HX106 displayed the slowest growth among the tested strains (Fig. S11 A). This underscores the synergetic effects of dual repression of *ppc* and *gltA* on 3M1B production per cell.

Furthermore, we targeted four additional genes—*acnB*, *ppsA*, *cpcB*, and *accC*—for repression, all of which demonstrated positive effects on IB and/or 3M1B production per cell. To investigate the impacts of multiplex repression, four new crRNA arrays containing three different protospacers were constructed to target *ppc*, *gltA*, and one of the aforementioned targets for repression. These arrays were introduced together with the dCas12a into strain HX11 to generate strains HX107, HX108, HX109, and HX110 (Table 1). As anticipated, strain HX107, with *ppc*, *gltA*, and *acnB* co-repressed, exhibited significantly improved IB/3M1B production per cell compared to the strain with *acnB* solely repressed (Fig. 6B). However, no significant difference was observed between strains HX106 and HX107 and similar IB/3M1B production per cell was achieved regardless of dual or triple repression (Fig. 6B). Although a slight improvement of IB/3M1B production per cell was achieved with single *acnB* repression as demonstrated in the preceding section, the positive effects of single *acnB* repression became negligible when incorporated into a single array with *ppc* and *gltA*. Comparable results were obtained for strains HX108, HX109, and HX110 (Fig. 6C–E). This phenomenon may be attributed to dilution effects when multiple protospacers are designed in a single crRNA array, potentially resulting in less efficient gene repression compared to individual crRNAs. Additionally, IB/3M1B production per cell in strain HX106 might correspond to a production threshold, which cannot be increased further in the cultivation system used in this study, even with a third gene repression.

On the other hand, PEP and pyruvate are two essential central carbon metabolites that are used as substrates for various cellular metabolic pathways, and *pyk* encoding pyruvate kinase is responsible for converting PEP into pyruvate, which is further used by the 2-keto acid pathway for IB/3M1B biosynthesis. Single repression of *ppc*, *ccmA*, and *ppsA* significantly enhanced IB/3M1B production per cell by increasing PEP pool to subsequently accumulate more pyruvate. Strain HX111 was generated with simultaneously repression of *ppc*, *ccmA*, and *ppsA*, with the aim to further channel more carbon towards PEP and pyruvate biosynthesis by downregulating three competing pathways simultaneously. Compared to the strains with *ccmA* or *ppsA* individually repressed, strain HX111 showed significantly improved IB/3M1B production per cell (Fig. 6F). However, no significant difference was observed between strains HX11-ppc and HX111 (Fig. 6F). Similarly, a single crRNA array that contains four protospacers was designed to simultaneously target *ppsA*, *accC*, and *pdh* (*sll1721* and *slr1934*), with the aim to channel less carbon source from pyruvate into PEP and acetyl-CoA. The resulting strain HX112 displayed slower growth rate and significantly enhanced IB/3M1B production per cell, compared to strains with single target gene repression (Fig. 6G, and Fig. S11G). The IB/3M1B production performance of HX112 implies that while individual gene knockdown may have a low impact in diverting metabolic flux, selective combinatorial multiplex repression of those genes could lead to substantial improvement in production. These findings highlight the effectiveness of simultaneous repression of multiple competing reactions that utilize central carbon metabolites as substrates to further enhance IB/3M1B production.

In summary, leveraging the CRISPRi-dCas12a system, we successfully targeted four genes simultaneously using a single crRNA array for the first time in cyanobacteria. Strain HX112 exhibited a synergistic effect of multiplex repression on IB/3M1B production per cell (Fig. 6G). Further attempts to simultaneously repress five targets using a single crRNA array were not successful due to the difficulty of plasmid construction. It is worth noting that the combination of multiple beneficial crRNAs did not consistently yield the anticipated synergistic effect in production enhancement (Fig. 6B–E). This underscores the notion that individually modulating the transcription level of each target gene may not always be optimal when combined in a production scenario. A combinatorial CRISPRi library approach [25] would be effective to allow for expression of multiple genes at different levels in a combination way to benefit IB/3M1B production in *Synechocystis*. In the present study, the identified strain HX106, which demonstrates the highest IB/3M1B production, shows significant potential to overcome the low titer limitations of cyanobacteria-based bioproduction. When cultivated in a photobioreactor with controlled conditions, HX106 could potentially achieve new records for photosynthetic IB/3M1B production. However, further research, including metabolomics analysis, is necessary to investigate how this strain alters its metabolism to accommodate elevated IB/3M1B biosynthesis.

## Conclusions

This work devised a dCas12a combinatorial multiplex repression system to attenuate reactions potentially competing with isobutanol and 3-methyl-1-butanol production in *Synechocystis* strain HX11. Upon cultivation optimization, repression of ten candidate genes significantly enhance IB/3M1B production. Notably, individual downregulation of *gltA* and *ppc* outperformed other targets. Moreover, synergetic effects on IB/3M1B production via simultaneous repression of four genes using a single crRNA array were achieved. This study introduces a novel approach for systematically fine-tuning metabolic pathways in *Synechocystis* to enhance IB/3M1B production, underscoring dCas12a as a promising tool for advancing biotechnological applications in cyanobacteria.

## Supplementary Information

Supplementary material 1.

## Data Availability

No datasets were generated or analysed during the current study.

## Abbreviations

3M1B: 3-Methyl-1-butanol
AccC: Acetyl-CoA carboxylase
Ach: Acetyl-CoA hydrolase
AcnB: Aconitase
AlsS: Acetolactate synthase
CcmA: 3-Deoxy-7-phosphoheptulonate synthase
CRISPRi: Clustered regularly interspaced short palindromic repeats interference
CpcB: Phycocyanin β-subunit
crRNA: CRISPR RNA
DAHP: 3-Deoxy-D-arabino-heptulosonate-7-phosphate
E4P: Erythrose-4-phosphate
*E. coli*: *Escherichia coli*
EPS: Extracellular polymeric substances
Fbp: Fructose-1,6-bisphosphate
FumC: Fumarase
GC: Gas chromatography
IB: Isobutanol
IlvC: Acetohydroxy-acid isomeroreductase
IlvD: Dihydroxy-acid dehydrase
IlvE: Branched-chain amino acid aminotransferase
Kivd: α-Ketoisovalerate decarboxylase
LB: Lysogeny broth
OD_750_: Optical density
PAM: Protospacer-adjacent motif
Pdh: Pyruvate dehydrogenase
PEP: Phosphoenolpyruvate
PhaA: Acetyl-CoA acetyltransferase
PHB: Poly-3-hydroxybutyrate
Pta: Phosphotransacetylase
RT-qPCR: Real-time quantitative PCR
Sps: Sucrose phosphate synthase
*Synechococcus*: *Synechococcus elongatus* PCC 7942
*Synechocystis*: *Synechocystis* Sp. PCC 6803
TCA cycle: Tricarboxylic acid cycle
TLA: Truncated light-harvesting antenna
