# Supplementary material for: dCas12a-mediated CRISPR interference for multiplex gene repression in cyanobacteria for enhanced isobutanol and 3-methyl-1-butanol production
Source: Microb Cell Fact. 2025 May 13;24:104. doi: 10.1186/s12934-025-02727-8 (PMC12076865; doi:10.1186/s12934-025-02727-8)
Supplement: Supplementary file 1 — Supplementary material 1. [file 12934_2025_2727_MOESM1_ESM.docx]

**dCas12a-mediated CRISPR interference for multiplex gene repression in cyanobacteria for enhanced isobutanol and 3-methyl-1-butanol production**

**Additional file 1**

**Fig. S1-11**

**Tables S1-2**

**
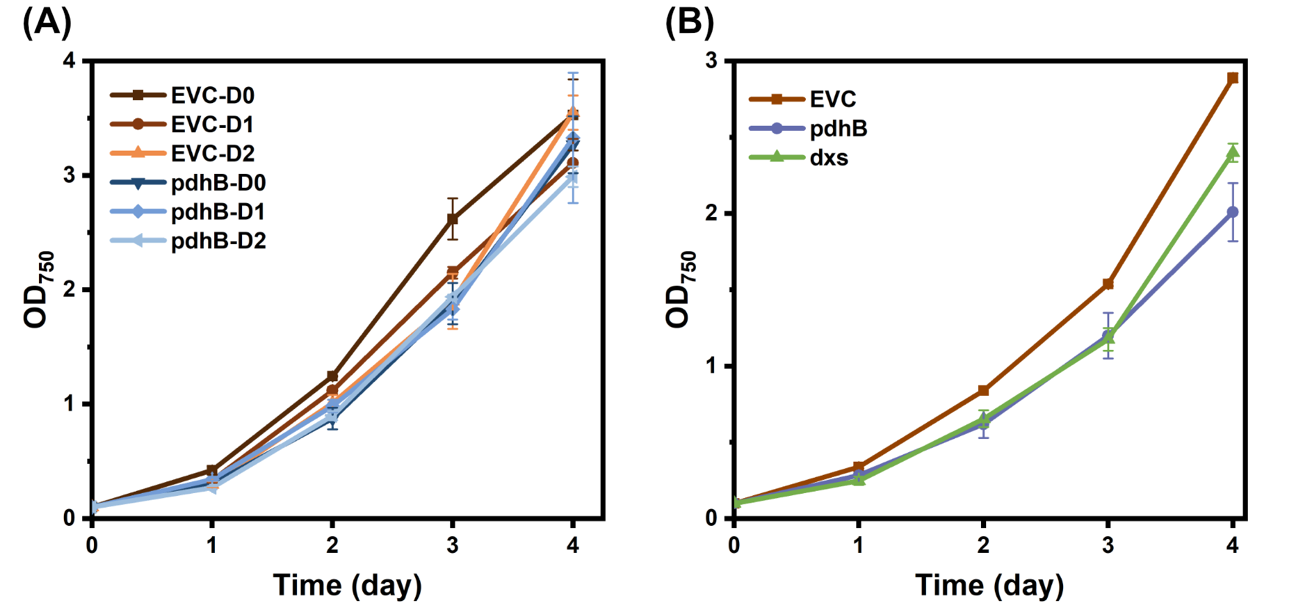
**

**Fig. S1:** Growth curves of cultures with CRISPRi tool induced with 3 mM rhamnose. EVC = empty vector control. Error bars represent standard deviation (three biology replicates).


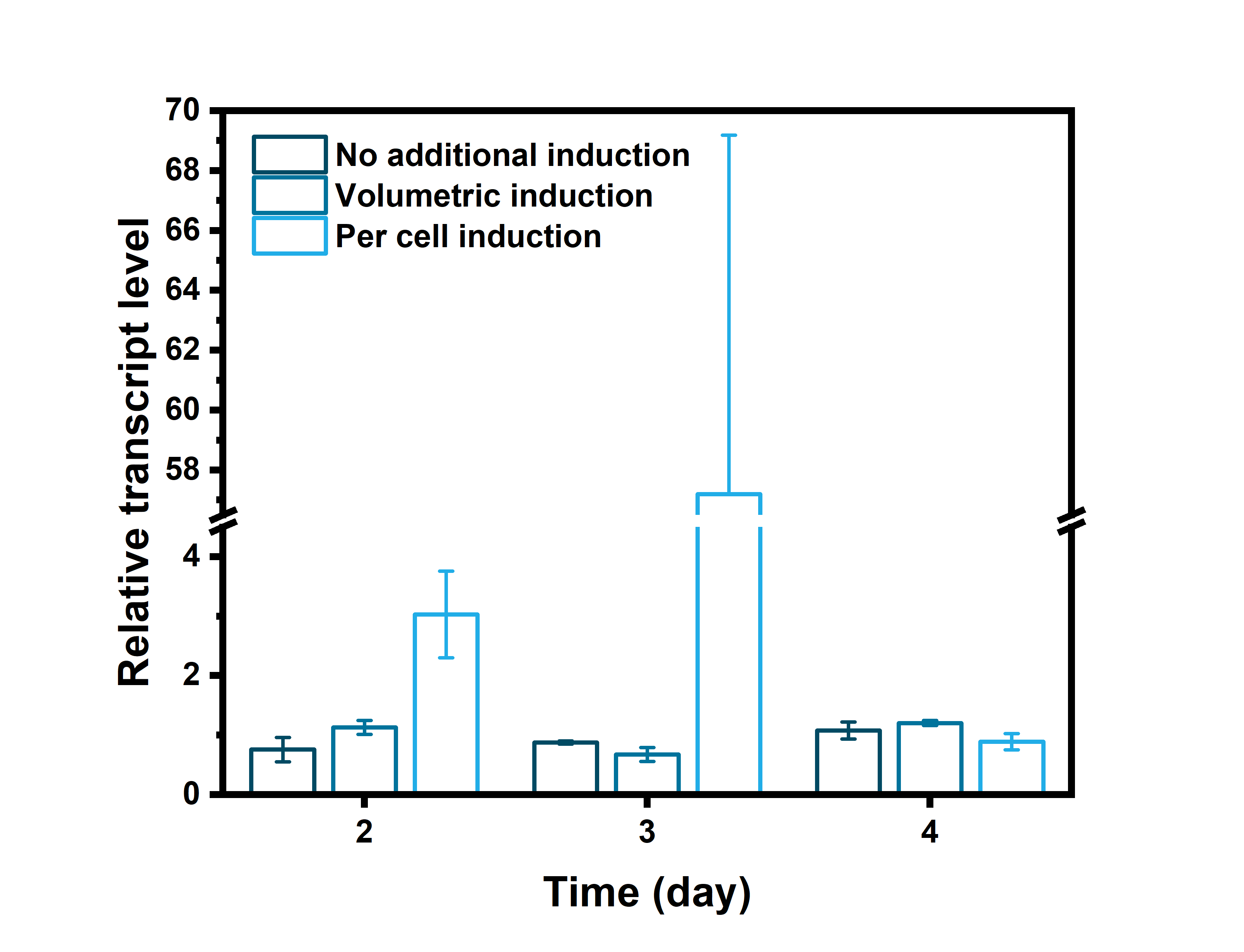


**Fig. S2:** *pdhB* downregulation induced with 3 mM rhamnose on day 0. No additional induction = rhamnose was added once at day 0. Volumetric induction = rhamnose was added a second time at day 2. 3 mM final concentration was added to the culture. Per cell induction = a second rhamnose induction was performed on day 2. Rhamnose concentration was normalised per cell. Error bars represent standard deviation (three biological replicates).


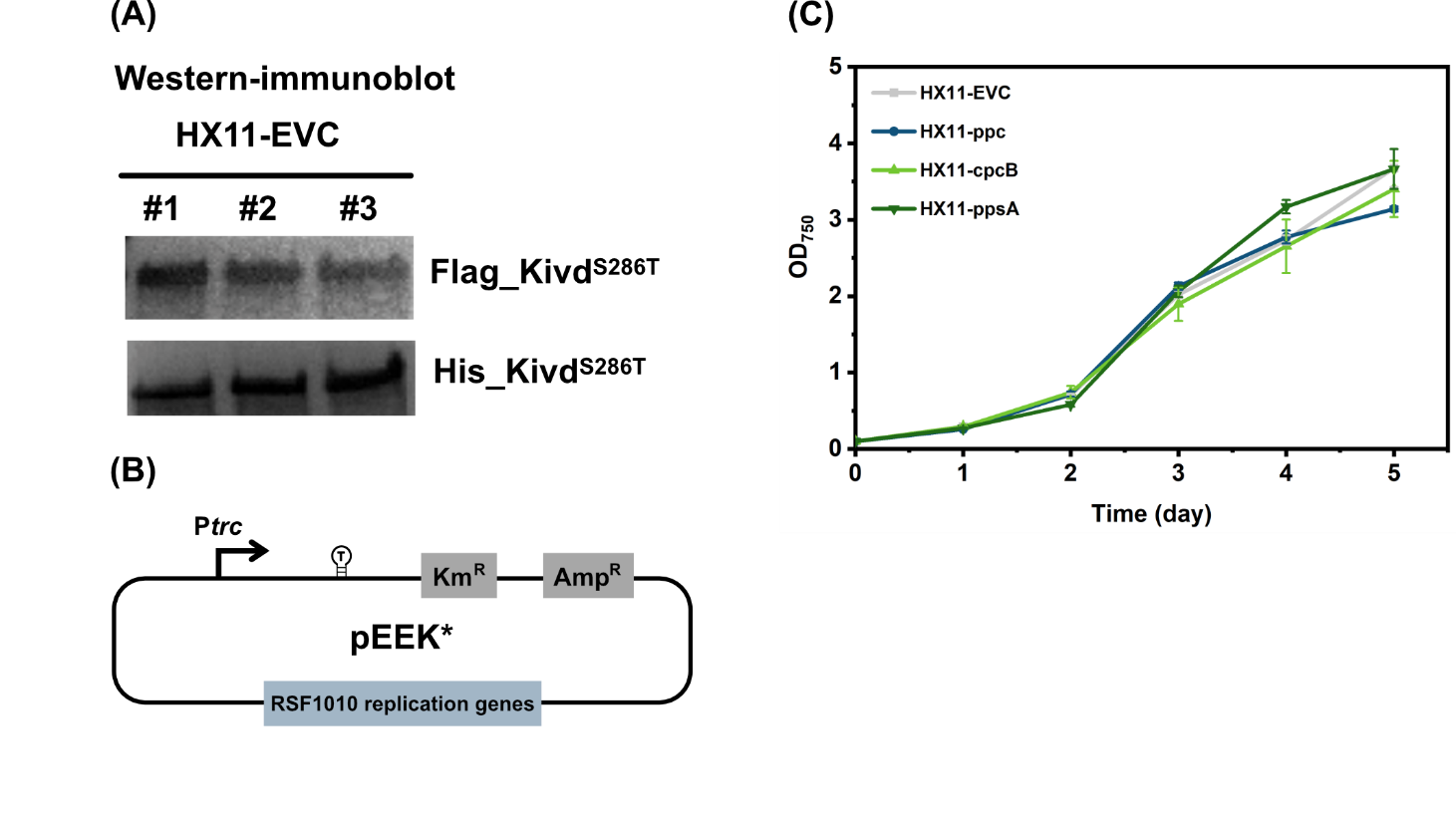


**Fig. S3:** Western-immunoblot analysis of strain HX11-EVC, schematic diagram of pEEK* plasmid, and growth profile of selected strains. **(A)** Western-immunoblot analysis expressed Kivd^S286T^ enzyme. Twenty micrograms of total soluble proteins were loaded to detect Flag-tagged and His-tagged proteins. Protein size: Kivd^S286T^, 61 kDa. The numbers represent biological triplicates. **(B)** Schematic diagram of pEEK* plasmid. It was used to generate strain HX11-pEEK* strain. **(C)** Growth profile of strains HX11-EVC, HX11-ppc, HX11-cpcB, and HX11-ppsA. Error bars represent standard deviation (two biological replicates).

**
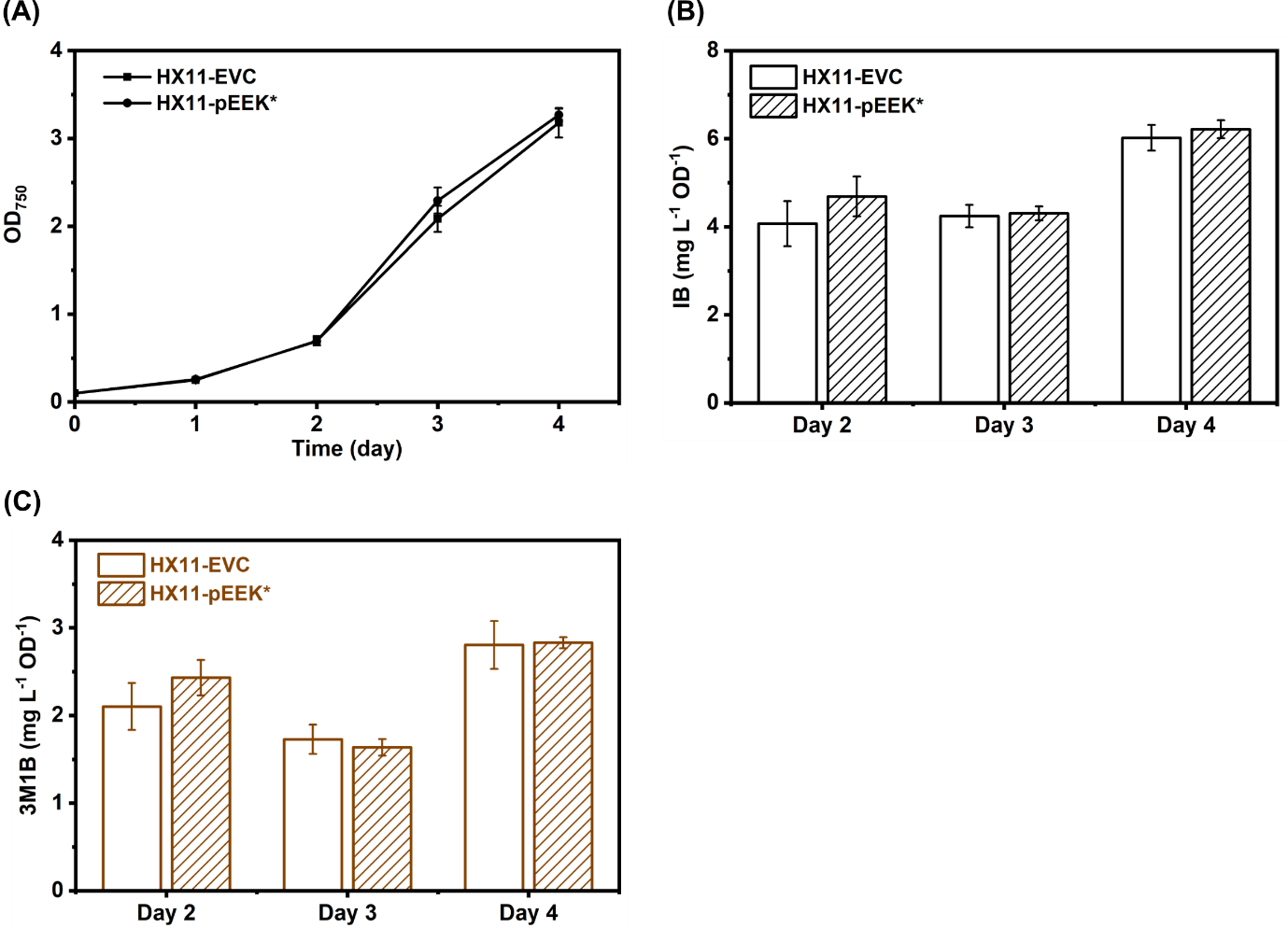
**

**Fig. S4:** Examination of the effects of dCas12a protein expression in isobutanol (IB) / 3-methyl-1-butanol (3M1B)-producing *Synechocystis* strain HX11. **(A)** Growth profile of strains HX11-EVC and HX11-pEEK*. **(B)** IB production per cell of strains HX11-EVC and HX11-pEEK* on days 2, 3, and 4. **(C)** 3M1B production per cell of strains HX11-EVC and HX11-pEEK* on days 2, 3, and 4. Results are the mean of three biological replicates, each with three technical replicates. Error bars represent standard deviation.

**
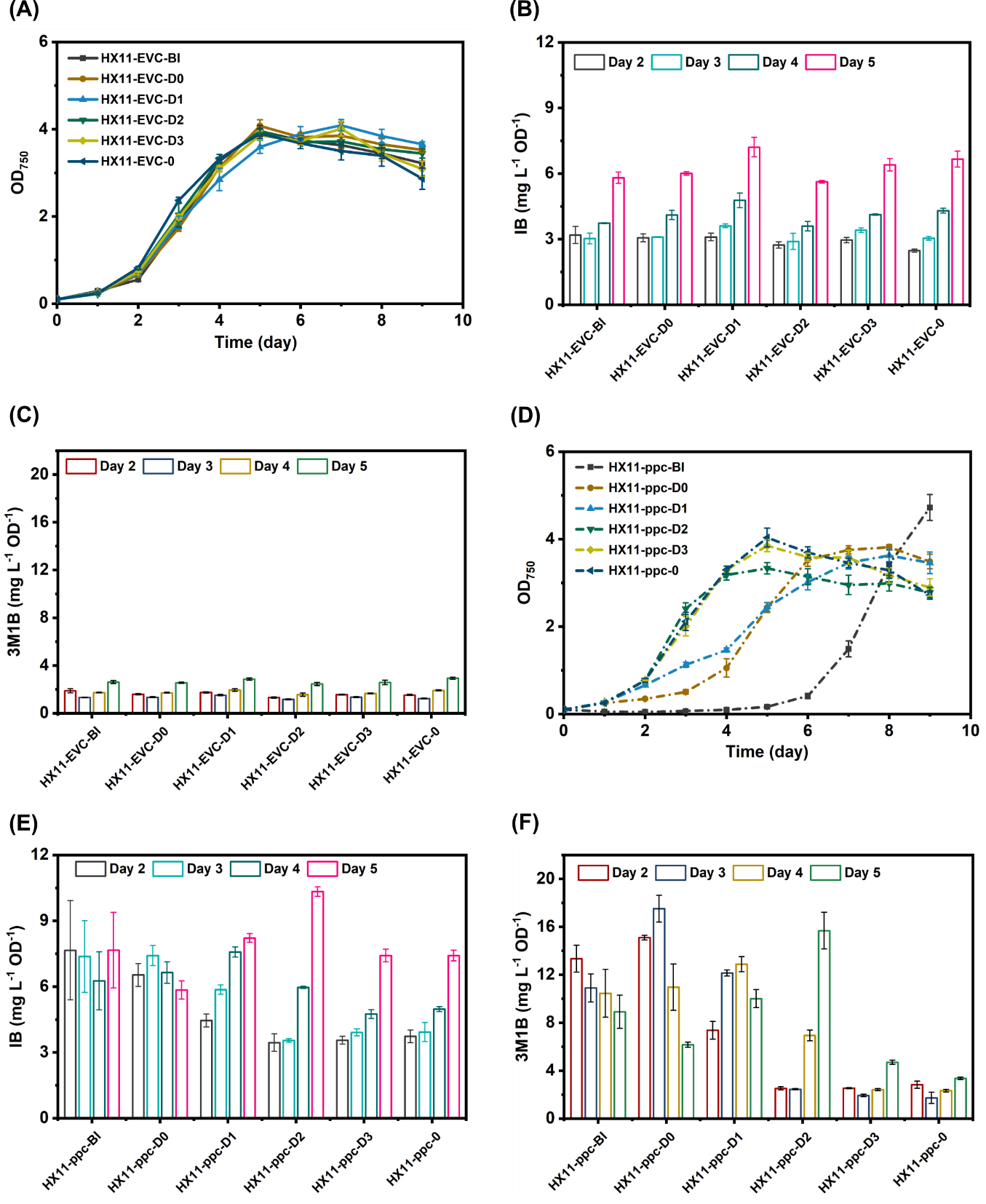
**

**Fig. S5:** Test of dCas12a-mediated CRISPRi system in isobutanol (IB) / 3-methyl-1-butanol (3M1B)-producing *Synechocystis* strain with varied rhamnose induction timepoints. Three millimolar rhamnose were added for induction in strains HX11-EVC and HX11-ppc before inoculation (BI), on days 0 (D0), 1 (D1), 2 (D2), 3 (D3), or without rhamnose addition (0). **(A)** Growth profile of strain HX11-EVC cultivated under varied induction timepoints. **(B)** IB production per cell of strain HX11-EVC with varied rhamnose induction timepoints on days 2, 3, 4, and 5. **(C)** 3M1B production per cell of strain HX11-EVC with varied rhamnose induction timepoints on days 2, 3, 4, and 5. **(D)** Growth profile of strain HX11-ppc cultivated under varied induction timepoints. **(E)** IB production per cell of strain HX11-ppc with varied rhamnose induction timepoints on days 2, 3, 4, and 5. **(F)** 3M1B production per cell of strain HX11-ppc with varied rhamnose induction timepoints on days 2, 3, 4, and 5. Results are the mean of three biological replicates. Error bars represent standard deviation.


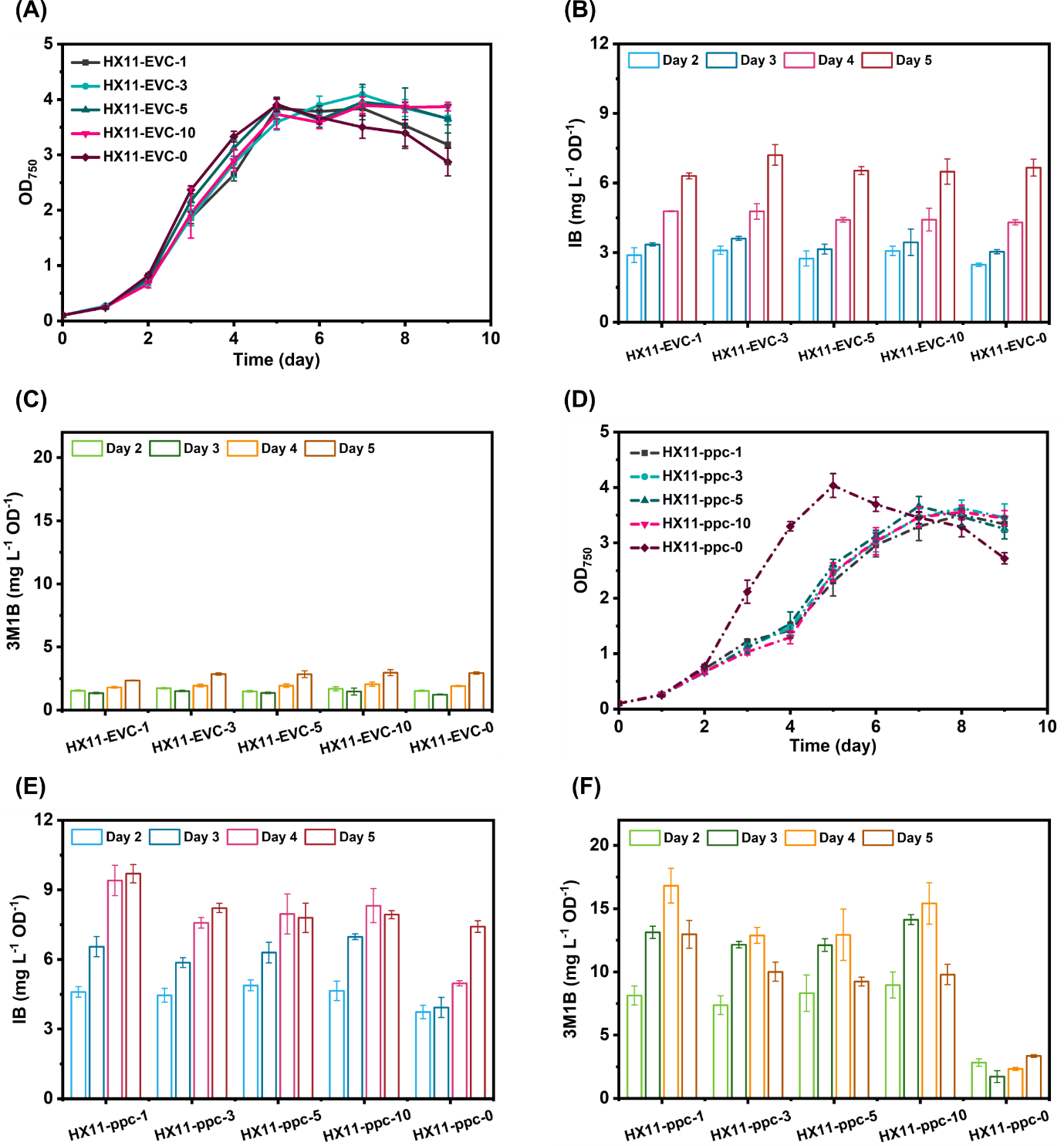


**Fig. S6:** Test of dCas12a-mediated CRISPRi system in isobutanol (IB) / 3-methyl-1-butanol (3M1B)-producing *Synechocystis* strain with varied rhamnose concentrations for induction. Rhamnose (0 mM, 1 mM, 3 mM, 5 mM, or 10 mM) was added for induction in strains HX11-EVC and HX11-ppc on day 1. **(A)** Growth profile of strain HX11-EVC cultivated under varied rhamnose concentrations. **(B)** IB production per cell of strain HX11-EVC with varied rhamnose concentrations for induction on days 2, 3, 4, and 5. **(C)** 3M1B production per cell of strain HX11-EVC with varied rhamnose concentrations for induction on days 2, 3, 4, and 5. **(D)** Growth profile of strain HX11-ppc cultivated under varied rhamnose concentrations. **(E)** IB production per cell of strain HX11-ppc with varied rhamnose concentrations for induction on days 2, 3, 4, and 5. **(F)** 3M1B production per cell of strain HX11-ppc with varied rhamnose concentrations for induction on days 2, 3, 4, and 5. Results are the mean of three biological replicates. Error bars represent standard deviation.


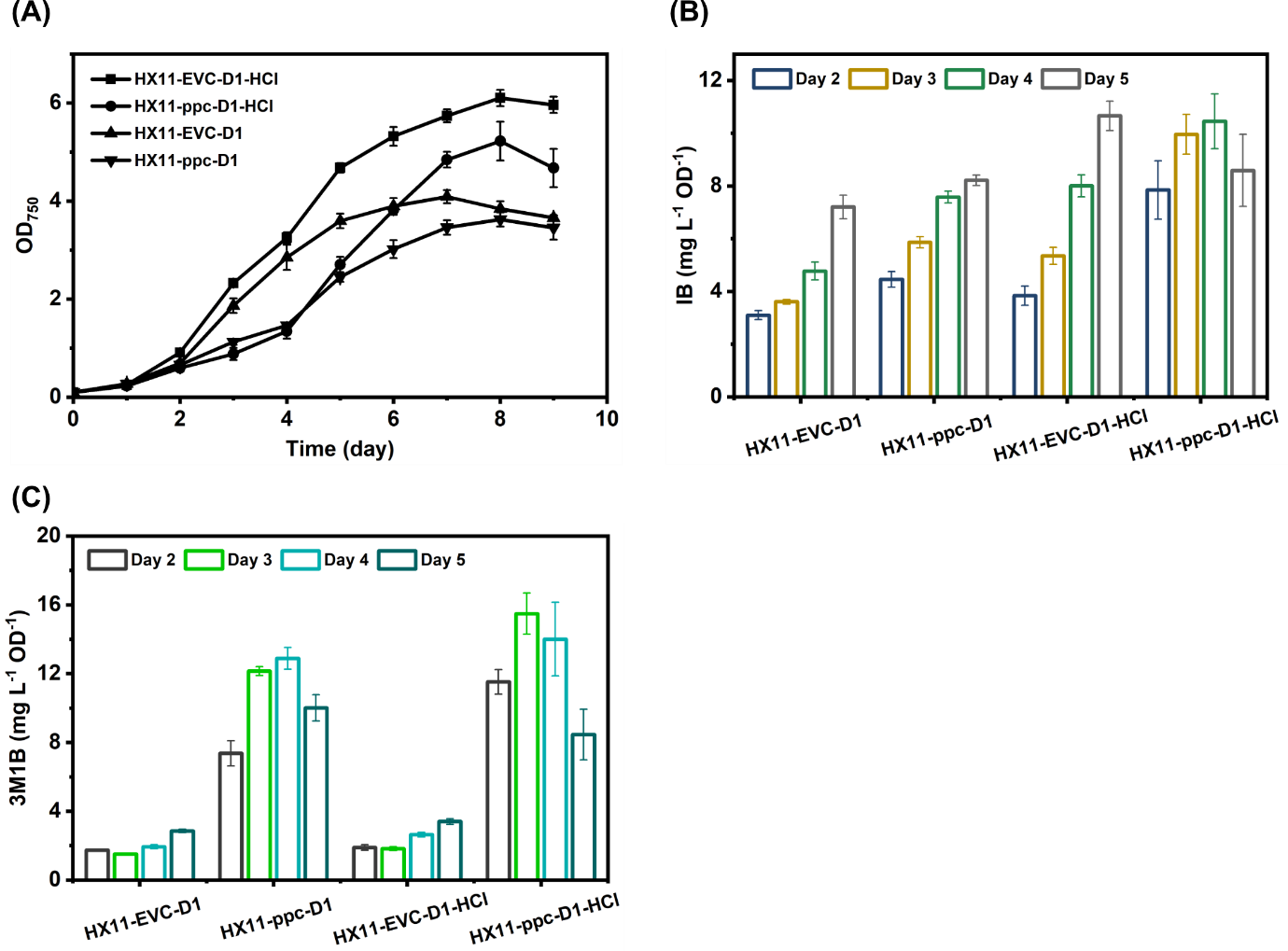


**Fig. S7:** Test of the effects of HCl titration on dCas12a-mediated CRISPRi system in isobutanol (IB) / 3-methyl-1-butanol (3M1B)-producing *Synechocystis* strain. Three millimolar rhamnose were added for induction in strains HX11-EVC and HX11-ppc on day 1 (D1). The target pH range of the cultures with HCl titration was between 7 and 8. **(A)** Growth profile of strains HX11-EVC and HX11-ppc cultivated with or without HCl titration. **(B)** IB production per cell of strains HX11-EVC and HX11-ppc cultivated with or without HCl titration on days 2, 3, 4, and 5. **(C)** 3M1B production per cell of strains HX11-EVC and HX11-ppc cultivated with or without HCl titration on days 2, 3, 4, and 5. Results are the mean of three biological replicates. Error bars represent standard deviation.

**
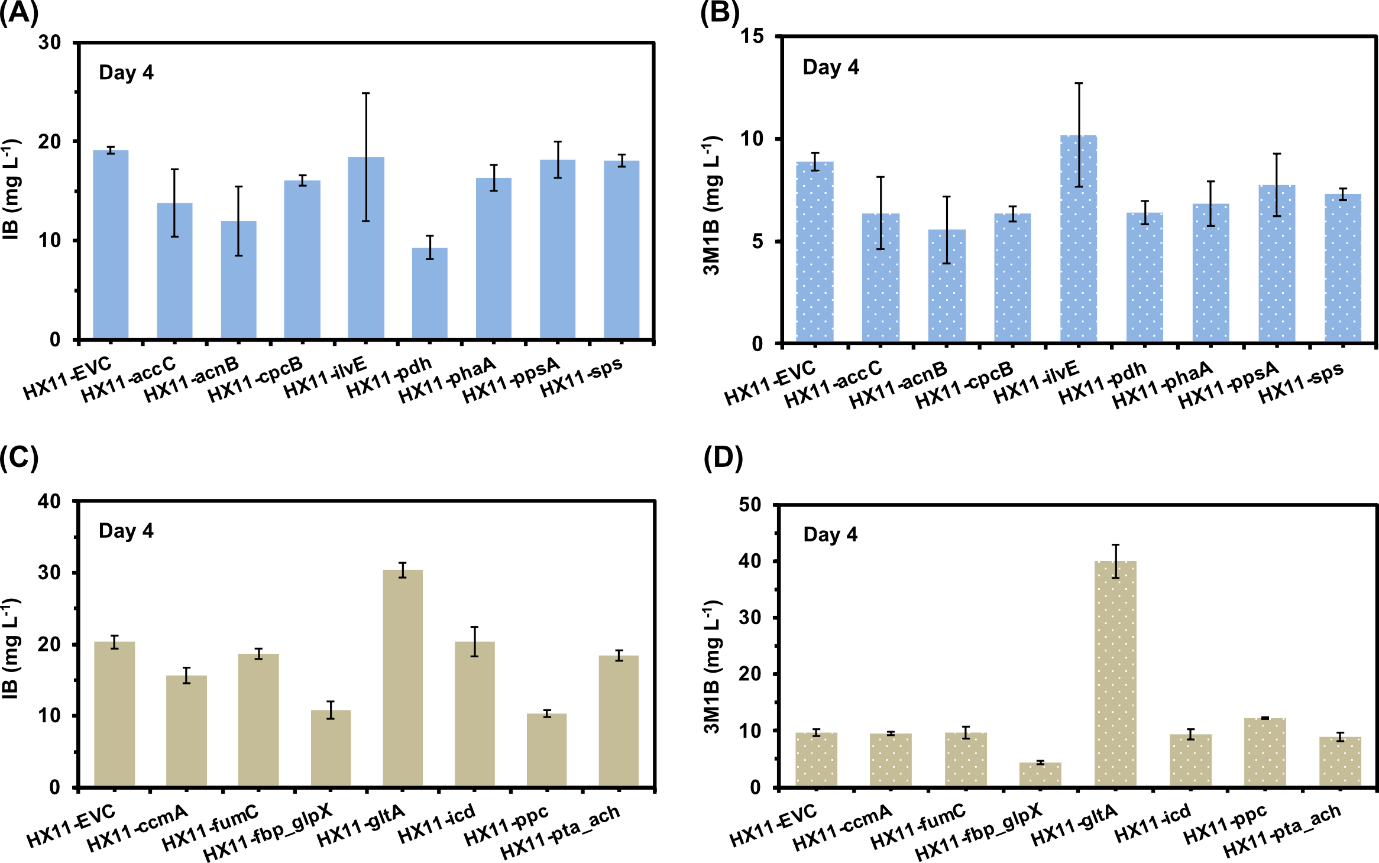
**

**Fig. S8:** Isobutanol **(**IB) and 3-methyl-1-butanol (3M1B) titers of engineered strains. **(A)** IB titer of the engineered *Synechocystis* strains with selected gene(s) repressed on day 4 (first batch). **(B)** 3M1B titer of the engineered *Synechocystis* strains with selected gene(s) repressed on day 4 (first batch). **(C)** IB titer of the engineered *Synechocystis* strains with selected gene(s) repressed on day 4 (second batch). **(D)** 3M1B titer of the engineered *Synechocystis* strains with selected gene(s) repressed on day 4 (second batch). Results are the mean of three biological replicates. Error bars represent standard deviation.

**
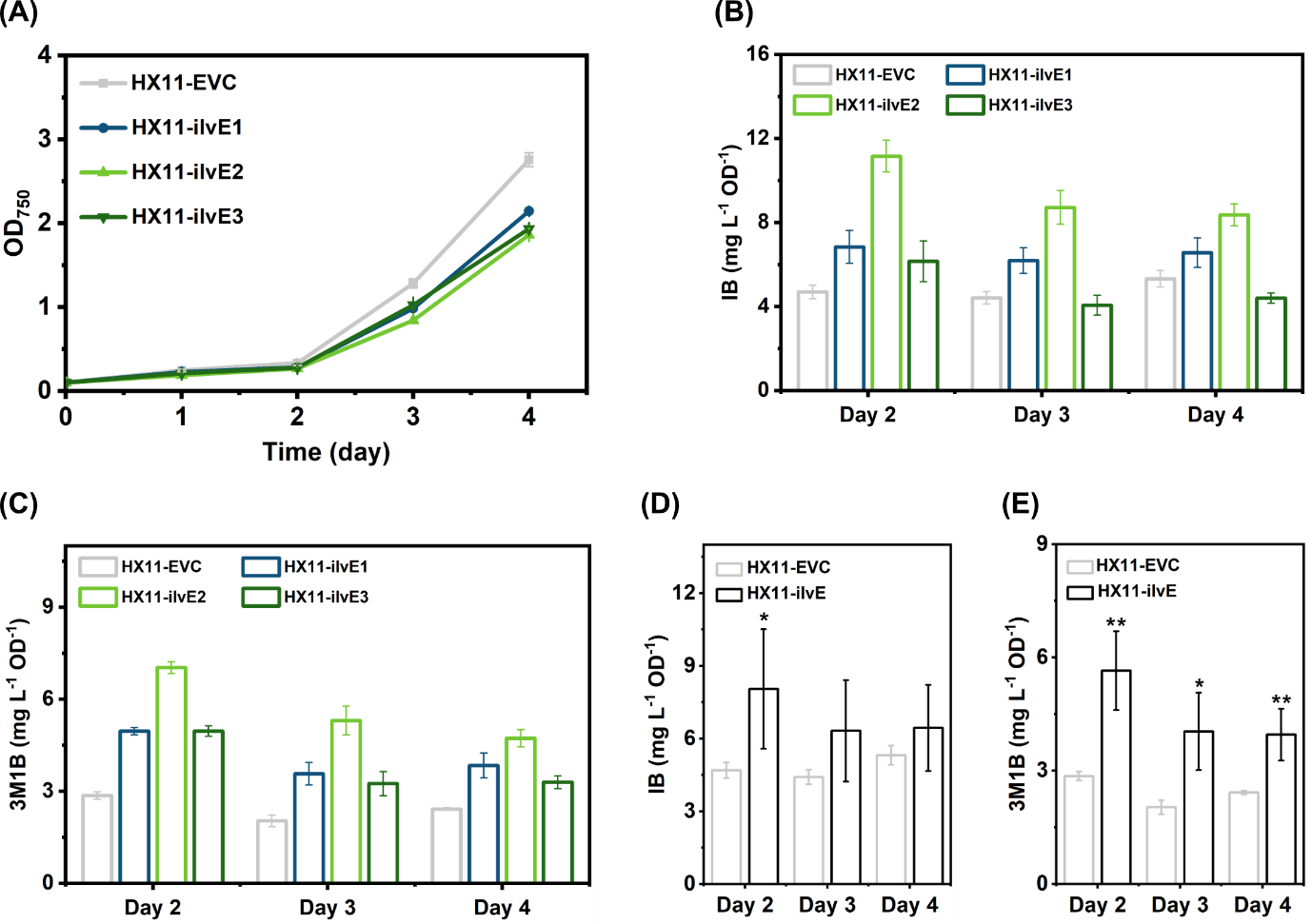
**

**Fig. S9:** Further investigation of HX11-ilvE strains. HX11-ilvE1, 2 and 3 represent three independent cell lines isolated after transformation. **(A)** Growth profile of strains HX11-EVC and HX11-ilvE1, 2 and 3. **(B)** IB production per cell of strains HX11-EVC and HX11-ilvE1, 2, and 3 on day 2, 3 and 4. **(C)** 3M1B production per cell of strains HX11-EVC and HX11-ilvE1, 2, and 3 on day 2, 3 and 4. **(D)** IB production per cell of strains HX11-EVC and HX11-ilvE on day 2, 3 and 4. **(E)** 3M1B production per cell of strains HX11-EVC and HX11-ilvE on day 2, 3 and 4. Error bars represent standard deviation (three biological replicates). Asterisk represents significant difference between recombinant strains and control strain (t-Test, *p < 0.05, **p < 0.005).

**
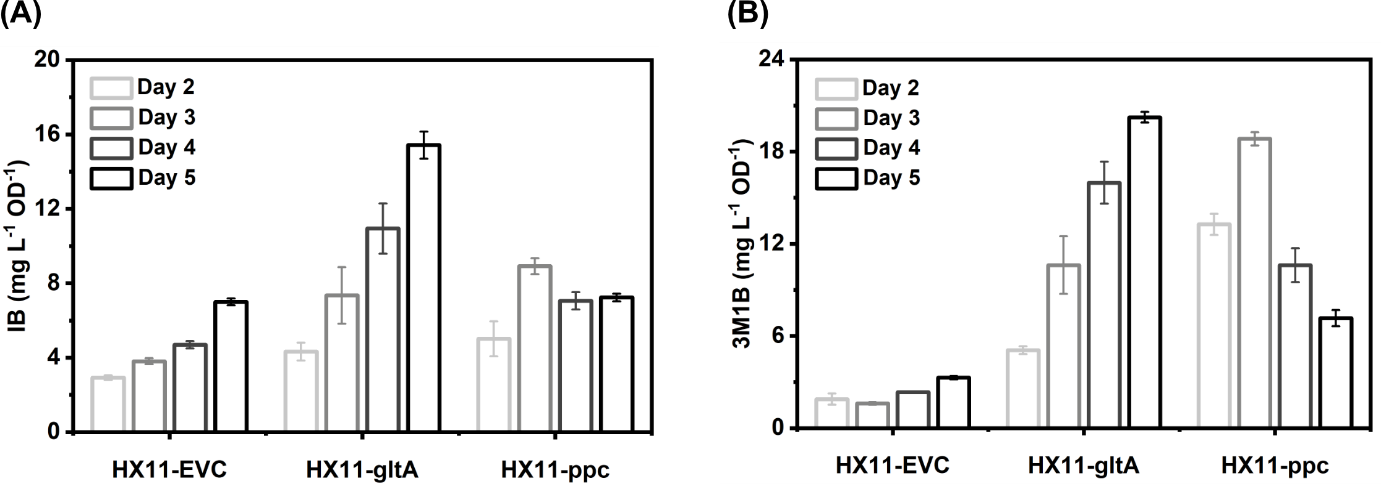
**

**Fig. S10:** Isobutanol **(**IB) and 3-methyl-1-butanol (3M1B) production per cell of selected strains. **(A)** IB production per cell of strains HX11-EVC, HX11-gltA, and HX11-ppc. **(B)** 3M1B production per cell of strains HX11-EVC, HX11-gltA, and HX11-ppc. Error bars represent standard deviation (three biological replicates).

**
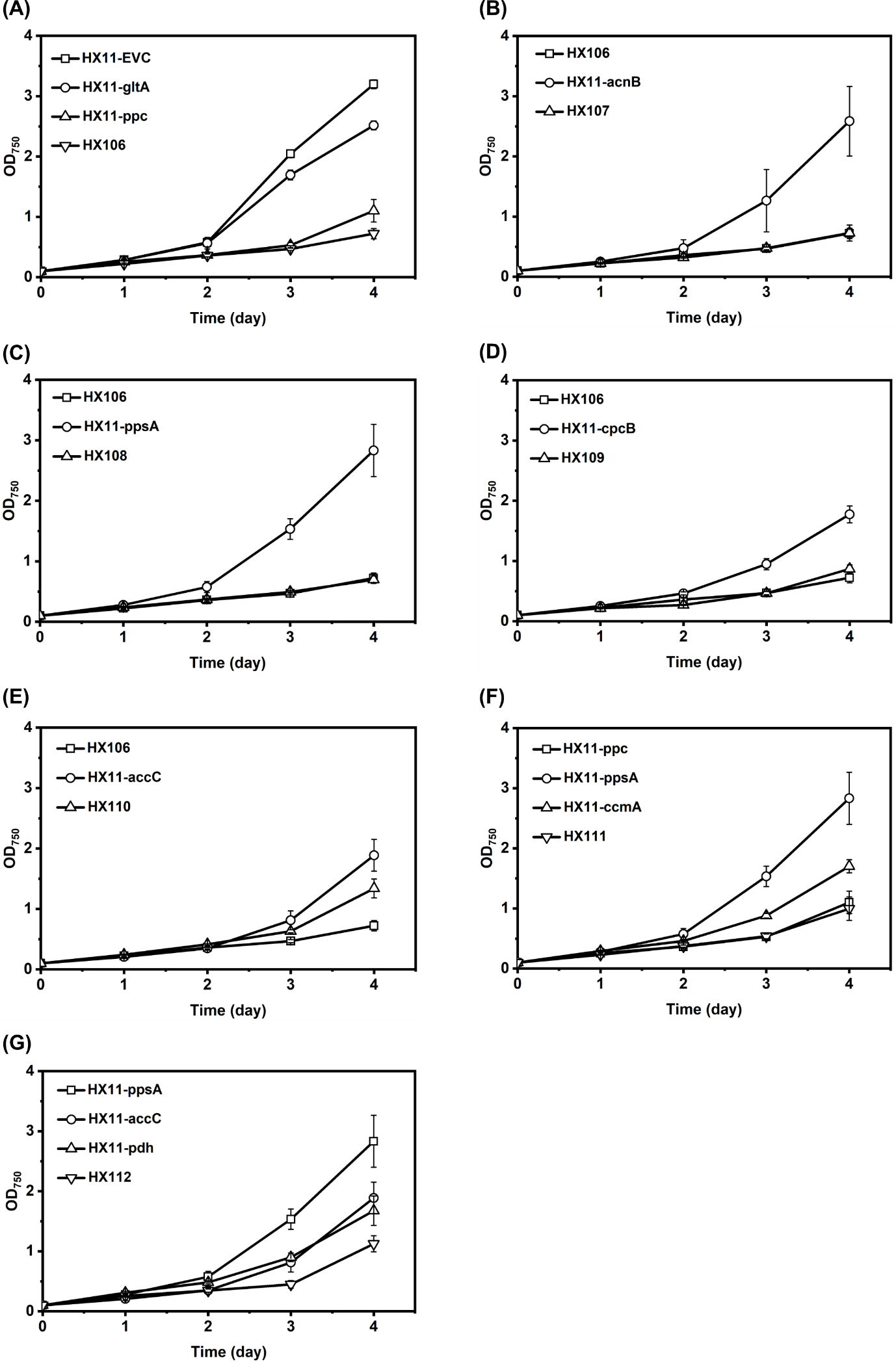
**

**Fig. S11:** Growth profiles of strains with combinatorial multiplex genes repression using dCas12a-mediated CRISPRi system for IB / 3M1B production. See Table 1 for the details of the strains. **(A)** Growth profile of strains HX11-EVC, HX11-gltA, HX11-ppc, and HX106. **(B)** Growth profile of strains HX106, HX11-acnB, and HX107. **(C)** Growth profile of strains HX106, HX11-ppsA, and HX108. **(D)** Growth profile of strains HX106, HX11-cpcB, and HX109. **(E)** Growth profile of strains HX106, HX11-accC, and HX110. **(F)** Growth profile of strains HX11-ppc, HX11-ppsA, HX11-ccmA, and HX111. **(G)** Growth profile of strains HX11-ppsA, HX11-accC, HX11-pdh, and HX112. Error bars represent standard deviation (three biological replicates).

**Table S1.** Plasmid used in this study. Expressed genes in bold.

| **Plasmid** | **Relevant characteristics^a^** | **Reference** |
| --- | --- | --- |
| pSL3287 | P*_trc2O_*_***dCas12***_T_J23119_***crRNA(no target)***_Km^R^ | (Knoot et al., 2020) |
| pSHDY_Prha:mVenus-PJ23119:rhaS | P*rhaBAD*_***mVenus***_T_J23119_rhaS_Km^R^ | (Behle et al., 2020) |
| pBB_dCas12a | P*rhaBAD*_***dCas12a***_T_P*rhaBAD*_***crRNA(no target)***_Km^R^ | This study |
| pBB1 | pBB_P*rhaBAD*_***dCas12a***_T_P*rhaBAD*_***crRNA(pdhB)***_Km^R^ | This study |
| pBB2 | pBB_P*rhaBAD*_***dCas12a***_T_P*rhaBAD*_***crRNA(dxs)***_Km^R^ | This study |
| pHX8 | pDdh_(P*trc*BCD_***kivd^S286T^***_T)_Cm^R^ | (Xie & Lindblad, 2022) |
| pHX15 | pSlr0168_(P*trc*BCD_***kivd^S286T^***_T)_Sp^R^ | (Xie & Lindblad, 2022) |
| pHX22 | pEEK* | Englund, E., not published |
| pHX23 | pBB_P*rhaBAD*_***dCas12a***_T_P*rhaBAD*_***crRNA(accC)***_Km^R^ | This study |
| pHX24 | pBB_P*rhaBAD*_***dCas12a***_T_P*rhaBAD*_***crRNA(acnB)***_Km^R^ | This study |
| pHX25 | pBB_P*rhaBAD*_***dCas12a***_T_P*rhaBAD*_***crRNA(ccmA)***_Km^R^ | This study |
| pHX26 | pBB_P*rhaBAD*_***dCas12a***_T_P*rhaBAD*_***crRNA(cpcB)***_Km^R^ | This study |
| pHX27 | pBB_P*rhaBAD*_***dCas12a***_T_P*rhaBAD*_***crRNA(fbp, glpX)***_Km^R^ | This study |
| pHX28 | pBB_P*rhaBAD*_***dCas12a***_T_P*rhaBAD*_***crRNA(fumC)***_Km^R^ | This study |
| pHX29 | pBB_P*rhaBAD*_***dCas12a***_T_P*rhaBAD*_***crRNA(gltA)***_Km^R^ | This study |
| pHX30 | pBB_P*rhaBAD*_***dCas12a***_T_P*rhaBAD*_***crRNA(icd)***_Km^R^ | This study |
| pHX31 | pBB_P*rhaBAD*_***dCas12a***_T_P*rhaBAD*_***crRNA(ilvE)***_Km^R^ | This study |
| pHX32 | pBB_P*rhaBAD*_***dCas12a***_T_P*rhaBAD*_***crRNA(sll1721, slr1934)***_Km^R^ | This study |
| pHX33 | pBB_P*rhaBAD*_***dCas12a***_T_P*rhaBAD*_***crRNA(phaA)***_Km^R^ | This study |
| pHX34 | pBB_P*rhaBAD*_***dCas12a***_T_P*rhaBAD*_***crRNA(ppc)***_Km^R^ | This study |
| pHX35 | pBB_P*rhaBAD*_***dCas12a***_T_P*rhaBAD*_***crRNA(ppsA)***_Km^R^ | This study |
| pHX36 | pBB_P*rhaBAD*_***dCas12a***_T_P*rhaBAD*_***crRNA(pta, ach)***_Km^R^ | This study |
| pHX37 | pBB_P*rhaBAD*_***dCas12a***_T_P*rhaBAD*_***crRNA(sps)***_Km^R^ | This study |
| pHX38 | pBB_P*rhaBAD*_***dCas12a***_T_P*rhaBAD*_***crRNA(ppc, gltA)***_Km^R^ | This study |
| pHX39 | pBB_P*rhaBAD*_***dCas12a***_T_P*rhaBAD*_***crRNA(ppc, gltA, acnB)***_Km^R^ | This study |
| pHX40 | pBB_P*rhaBAD*_***dCas12a***_T_P*rhaBAD*_***crRNA(ppc, gltA, ppsA)***_Km^R^ | This study |
| pHX41 | pBB_P*rhaBAD*_***dCas12a***_T_P*rhaBAD*_***crRNA(ppc, gltA, cpcB)***_Km^R^ | This study |
| pHX42 | pBB_P*rhaBAD*_***dCas12a***_T_P*rhaBAD*_***crRNA(ppc, gltA, accC)***_Km^R^ | This study |
| pHX43 | pBB_P*rhaBAD*_***dCas12a***_T_P*rhaBAD*_***crRNA(ppc, ppsA, ccmA)***_Km^R^ | This study |
| pHX44 | pBB_P*rhaBAD*_***dCas12a***_T_P*rhaBAD*_***crRNA(ppsA, accC, sll1721, slr1934)***_Km^R^ | This study |
| pHX45 | pBB_P*rhaBAD*_***dCas12a***_T_P*rhaBAD*_***crRNA(pdhB)***_Km^R^ | This study |
| pHX46 | pBB_P*rhaBAD*_***dCas12a***_T_P*rhaBAD*_***crRNA(dxs)***_Km^R^ | This study |

a Km^R^, Kanamycin resistance cassette; Sp^R^, spectinomycin resistance cassette; Cm^R^: chloramphenicol resistance cassette; T, Terminator.

**Table S2.** Oligonucleotides used in this study.

| **Primer name** | **Oligonucleotides sequence** |
| --- | --- |
| **A. Primers for fragment amplification** | |
| RhaS_F | ATATATGGTACCTTGACAGCTAGCTCAGTCCT |
| RhaS_R | TGTTCACAATTTGCTGAATTGTGGCTCAACTCGTATAAACGCAGAA |
| Prha_F | GCCTTTCTGCGTTTATACGAGTTGAGCCACAATTCAGCAAATTGT |
| Prha_R | ATATATGGTACCTTCATTACGACCAGTCTAAA |
| dCas12_F | ATATATACTAGTGTCCTAAATACTATCTTACAAAT |
| dCas12_R | CGCTTTTTAGACTGGTCGTAATGAAGCTGATTTAGGCAAAAACG |
| Prha_gRNA_F | ATATATGTCGACGCCACAATTCAGCAAATTGT |
| Prha_gRNA_R | TAGACCCGTTTTTGCCTAAATCAGCTTCATTACGACCAGTCTAAA |
| **B. Primers for oligo annealing** | |
| g_pdhB_F(P) | Phos-AGATTTGTGCCCCGATCGCCCTTG |
| g_pdhB_R(P) | Phos- AGACCAAGGGCGATCGGGGCACAA |
| g_dxs_F(P) | Phos-AGATTCCAGACTGTGACTGGGGTT |
| g_dxs_R(P) | Phos-AGACAACCCCAGTCACAGTCTGGA |
| g_acnB_F(P) | Phos-AGATACCATGAACTAATATTGCCG |
| g_acnB_R(P) | Phos-AGACCGGCAATATTAGTTCATGGT |
| g_accC_F(P) | Phos-AGATTACTAAAAGACTAAACACAT |
| g_accC_R(P) | Phos-AGACATGTGTTTAGTCTTTTAGTA |
| g_ccmA_F(P) | Phos-AGATGCATCGGCTTTTCGAGTCCG |
| g_ccmA_R(P) | Phos-AGACCGGACTCGAAAAGCCGATGC |
| g_cpcB_F(P) | Phos-AGATAGAAAATCCCAACTCATAAA |
| g_cpcB_R(P) | Phos-AGACTTTATGAGTTGGGATTTTCT |
| g_fbp_glpX_F(P) | Phos-AGATAGCCTAGCAATCCAAGGAGAGTCTAAGAACTTTAAATAATTTCTACTGTTGTAGATA CACTTAATCACAAGCTAGG |
| g_fbp_glpX_R(P) | Phos-AGACCCTAGCTTGTGATTAAGTGTATCTACAACAGTAGAAATTATTTAAAGTTCTTAGACT  CTCCTTGGATTGCTAGGCT |
| g_fumC_F(P) | Phos-AGATCCACCGGAGTATTTGCAAAT |
| g_fumC_R(P) | Phos-AGACATTTGCAAATACTCCGGTGG |
| g_gltA_F(P) | Phos-AGATTCCGTTATAGGTGAATTATA |
| g_gltA_R(P) | Phos-AGACTATAATTCACCTATAACGGA |
| g_icd_F(P) | Phos-AGATTGATCTGCTCCGTGCCGTTT |
| g_icd_R(P) | Phos-AGACAAACGGCACGGAGCAGATCA |
| g_ilvE_F(P) | Phos-AGATGATGACCGGGGTTAAGTCCA |
| g_ilvE_R(P) | Phos-AGACTGGACTTAACCCCGGTCATC |
| g_pdh_F(P) | Phos-AGATTTGTGCCCCGATCGCCCTTGGTCTAAGAACTTTAAATAATTTCTACTGTTGTAGATC  CCTTTATCTACTGCAAATT |
| g_pdh_R(P) | Phos-AGACAATTTGCAGTAGATAAAGGGATCTACAACAGTAGAAATTATTTAAAGTTCTTAGACC  AAGGGCGATCGGGGCACAA |
| g_phaA_F(P) | Phos-AGATAGCTGGAGGCGGAGTTAATG |
| g_phaA_R(P) | Phos-AGACCATTAACTCCGCCTCCAGCT |
| g_ppc_F(P) | Phos-AGATTACAGTAGCAACGCATTCTG |
| g_ppc_R(P) | Phos-AGACCAGAATGCGTTGCTACTGTA |
| g_ppsA_F(P) | Phos-AGATGGGGTAAATAGATATGGTAA |
| g_ppsA_R(P) | Phos-AGACTTACCATATCTATTTACCCC |
| g_pta_ach_F(P) | Phos-AGATCTGGATACGTTGAGGTTATTGTCTAAGAACTTTAAATAATTTCTACTGTTGTAGATAT  GGGGGAAACAGCTTCACT |
| g_pta_ach_R(P) | Phos-AGACAGTGAAGCTGTTTCCCCCATATCTACAACAGTAGAAATTATTTAAAGTTCTTAGACA  ATAACCTCAACGTATCCAG |
| g_sps_F(P) | Phos-AGATTAGTAATTTGTAAAACTTAT |
| g_sps_R(P) | Phos-AGACATAAGTTTTACAAATTACTA |
| g_ppc_gltA_F(P) | Phos-AGATTACAGTAGCAACGCATTCTGGTCTAAGAACTTTAAATAATTTCTACTGTTGTAGATT  CCGTTATAGGTGAATTATA |
| g_PEPc_gltA_R(P) | Phos-AGACTATAATTCACCTATAACGGAATCTACAACAGTAGAAATTATTTAAAGTTCTTAGACC  AGAATGCGTTGCTACTGTA |
| g_PEPc_gltA_F1(P) | Phos-AGATTACAGTAGCAACGCATTCTGGTCTAAGAACTTTAAATAATTTCTACTGTTGTAGATT  CCGTTATAGGTGAATTATA |
| g_PEPc_gltA_R1(P) | Phos-ATCTACAACAGTAGAAATTATTTAAAGTTCTTAGACCAGAATGCGTTGCTACTGTA |
| g_acnB_F1(P) | Phos-GTCTAAGAACTTTAAATAATTTCTACTGTTGTAGATACCATGAACTAATATTGCCG |
| g_acnB_R1(P) | Phos-AGACCGGCAATATTAGTTCATGGTATCTACAACAGTAGAAATTATTTAAAGTTCTTAGACT  ATAATTCACCTATAACGGA |
| g_ppsA_F1(P) | Phos-GTCTAAGAACTTTAAATAATTTCTACTGTTGTAGATGGGGTAAATAGATATGGTAA |
| g_ppsA_R1(P) | Phos-AGACTTACCATATCTATTTACCCCATCTACAACAGTAGAAATTATTTAAAGTTCTTAGACTA  TAATTCACCTATAACGGA |
| g_cpcB_F1(P) | Phos-GTCTAAGAACTTTAAATAATTTCTACTGTTGTAGATAGAAAATCCCAACTCATAAA |
| g_cpcB_R1(P) | Phos-AGACTTTATGAGTTGGGATTTTCTATCTACAACAGTAGAAATTATTTAAAGTTCTTAGACTA  TAATTCACCTATAACGGA |
| g_accC_F1(P) | Phos-GTCTAAGAACTTTAAATAATTTCTACTGTTGTAGATTACTAAAAGACTAAACACAT |
| g_accC_R1(P) | Phos-AGACATGTGTTTAGTCTTTTAGTAATCTACAACAGTAGAAATTATTTAAAGTTCTTAGACTA  TAATTCACCTATAACGGA |
| g_ccmA_F1(P) | Phos-GTCTAAGAACTTTAAATAATTTCTACTGTTGTAGATGCATCGGCTTTTCGAGTCCG |
| g_ccmA_R1(P) | Phos-AGACCGGACTCGAAAAGCCGATGCATCTACAACAGTAGAAATTATTTAAAGTTCTTAGAC  TTACCATATCTATTTACCCC |
| g_ppsA_accC_F1(P) | Phos-AGATGGGGTAAATAGATATGGTAAGTCTAAGAACTTTAAATAATTTCTACTGTTGTAGATT  ACTAAAAGACTAAACACAT |
| g_ppsA_accC_R1(P) | Phos-ATCTACAACAGTAGAAATTATTTAAAGTTCTTAGACTTACCATATCTATTTACCCC |
| g_sll1721_F1(P) | Phos-GTCTAAGAACTTTAAATAATTTCTACTGTTGTAGATTTGTGCCCCGATCGCCCTTG |
| g_sll1721_R1(P) | Phos-ATCTACAACAGTAGAAATTATTTAAAGTTCTTAGACATGTGTTTAGTCTTTTAGTA |
| g_slr1934_F1(P) | Phos-GTCTAAGAACTTTAAATAATTTCTACTGTTGTAGATCCCTTTATCTACTGCAAATT |
| g_slr1934_R1(P) | Phos-AGACAATTTGCAGTAGATAAAGGGATCTACAACAGTAGAAATTATTTAAAGTTCTTAGACC  AAGGGCGATCGGGGCACAA |
| **C. Primers for *E. coli* colony PCR and *Synechocystis* colony PCR** | |
| dCas12_Seq6 | AGCAGATGTAAATGGCAATTTCTTTG |
| g_accC_SR | TACTAAAAGACTAAACACATGTCTAAG |
| g_acnB_SR | GTTGTAGATACCATGAACTAATATTGCCG |
| g_ccmA_SR | GCATCGGCTTTTCGAGTCCG |
| g_cpcB_SR | GTTGTAGATAGAAAATCCCAACTCATAAAG |
| g_fbp_SR | AGCCTAGCAATCCAAGGAGA |
| g_fumC_SR | GATCCACCGGAGTATTTGCAAAT |
| g_gltA_SR | GTAGATTCCGTTATAGGTGAATTATAGTC |
| g_icd_SR | GATCTGCTCCGTGCCGTTTG |
| g_ilvE_SR | GATGACCGGGGTTAAGTCCA |
| g_slr1934_SR | CCCTTTATCTACTGCAAATTGTC |
| g_phaA_SR | AGCTGGAGGCGGAGTTAATG |
| g_ppc_SR | GATTACAGTAGCAACGCATTCTG |
| g_ppsA_SR | GTTGTAGATGGGGTAAATAGATATGGTAAG |
| g_pta_SR | GTAGATCTGGATACGTTGAGGTTATT |
| g_sps_SR | AGATTAGTAATTTGTAAAACTTATGTCTAAGAAC |
| VF2 | TGCCACCTGACGTCTAAGAA |
| VR | ATTACCGCCTTTGAGTGAGC |
| dCas12_fwd | GTCCTAAATACTATCTTACAAAT |
| KanR_fwd | CTGCCTCGGTGAGTTTTCTC |
| **D. Primers for RT-qPCR** | |
| qrnpB_F | CGTTAGGATAGTGCCACAG |
| qrnpB_R | CGCTCTTACCGCACCTTTG |
| qpdhB_F | CTTCCCAAGATATTCCCACTCC |
| qpdhB_R | GCCGATGATCGCCTTAACT |
| qdxs_F | CCCATACCAGACTAATGGTGATT |
| qdxs_R | TGCTGAGGCGGACTTTATTT |
| qcpcB_F | GACGTATTCACTCGGGTTGTT |
| qcpcB_R | CAATCCGTTTGTTGCCTTCAG |
| qPEPc_F | CGAAGAACTTTCTCTCCGCTATC |
| qPEPc_R | CTGGGTTGGCTAGACGATTATT |
| qppsA_F | TATTCACTGACCGGGCTATTTC |
| qppsA_R | AGATCAGAACGCACCATCTTT |

**References**

Behle, A., Saake, P., Germann, A. T., Dienst, D., & Axmann, I. M. (2020, Apr 17). Comparative Dose-Response Analysis of Inducible Promoters in Cyanobacteria. *ACS Synth Biol, 9*(4), 843-855. <https://doi.org/10.1021/acssynbio.9b00505>

Knoot, C. J., Biswas, S., & Pakrasi, H. B. (2020, Jan 17). Tunable Repression of Key Photosynthetic Processes Using Cas12a CRISPR Interference in the Fast-Growing Cyanobacterium Synechococcus sp. UTEX 2973. *ACS Synth Biol, 9*(1), 132-143. <https://doi.org/10.1021/acssynbio.9b00417>

Xie, H., & Lindblad, P. (2022, Feb 1). Expressing 2-keto acid pathway enzymes significantly increases photosynthetic isobutanol production. *Microb Cell Fact, 21*(1), 17. <https://doi.org/10.1186/s12934-022-01738-z>
